# Synthesis of PtCu/C Nanostructured Electrocatalysts for the Oxygen Reduction Reaction via One-Step Electrochemical Erosion

**DOI:** 10.1021/acsami.5c22270

**Published:** 2026-01-06

**Authors:** Peter M. Schneider, Eva Kolíbalová, Jhonatan Rodriguez-Pereira, Theophilus K. Sarpey, Christian M. Schott, Elena L. Gubanova, Pavan Kumar Chennam, Anatoliy Senyshyn, Christine Benning, Martin Elsner, Jan M. Macak, Aliaksandr S. Bandarenka

**Affiliations:** † Physics of Energy Conversion and Storage, Technical University of Munich, James-Franck-Str. 1, 85748 Garching, Germany; ‡ Central European Institute of Technology, 232848 Brno University of Technology, Purkynova 123, 61200 Brno, Czech Republic; § Center of Materials and Nanotechnologies, 48252 University of Pardubice, Nam. Cs. Legii 565, 53002 Pardubice, Czech Republic; ∥ Materials Research Department, GSI Helmholtzzentrum für Schwerionenforschung GmbH, Planckstraße 1, 64291 Darmstadt, Germany; ⊥ Heinz Maier-Leibnitz Zentrum (MLZ), Technische Universität München, Lichtenbergstr. 1, 85748 Garching, Germany; # Chair of Analytical Chemistry and Water Chemistry, School of Natural Sciences, Technical University of Munich, Lichtenbergstr. 4, 85748 Garching, Germany; ¶ 9184 Catalysis Research Center TUM, Ernst-Otto-Fischer-Str. 1, 85748 Garching, Germany

**Keywords:** oxygen reduction reaction, nanoparticles, electrochemical erosion, platinum alloys, electrocatalysis

## Abstract

Reducing the precious metal loading while increasing the oxygen reduction reaction (ORR) mass activity of novel electrocatalysts constitutes one of the remaining key challenges in the widespread application of proton exchange membrane fuel cells, which is inevitable for the transition to the climate-neutral hydrogen economy. However, this requires a simple, scalable, and affordable production of active nanostructured electrocatalysts. Alloyed nanoparticles of Platinum (Pt) with transition metals like cobalt, nickel, or copper have shown promising activity toward ORR, but their preparation usually involves complex multistep processes and environmentally harmful surfactants or structure-capping agents. In this work, we present the successful synthesis of nonspherical copper-alloyed Pt nanoparticles (PtCu) by employing a simple one-step top-down approach without surfactants or capping agents. The electrocatalysts were characterized by high-resolution transmission electron microscopy, energy-dispersive X-ray spectroscopy, X-ray diffraction, X-ray photoelectron spectroscopy, and inductively coupled plasma mass spectrometry. The ORR kinetics were evaluated using the rotating (ring) disk electrode technique. The synthesized PtCu/C catalysts revealed outstanding mass activities of ∼1.2 A mg_Pt_^–1^ at 0.9 V vs the reversible hydrogen electrode, which clearly surpasses state-of-the-art Pt-based catalysts in the literature and demonstrates the highest ORR mass activities reported for PtCu nanoparticles.

## Graphical abstract

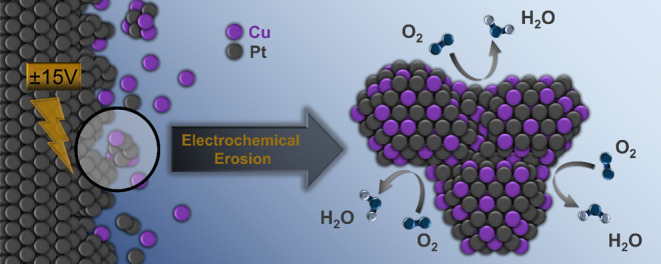

## Introduction

As the global energy demand steadily increases, the responsibility to cut harmful greenhouse gas emissions is growing. This can only be achieved by decoupling the global energy consumption from carbonous energy carriers to minimize the consequences of climate change. Hydrogen offers great potential for a future, carbon-free energy cycle due to its high mass-specific energy density, obtained through renewable energy-powered electrolysis and used as a fuel in, for example, proton exchange membrane fuel cells (PEMFCs).^1^ The oxygen reduction reaction (ORR) at the cathode side of PEMFCs constitutes one of the most important energy-related reactions.^2^ Lots of research effort has been made to develop nanostructured electrocatalysts to improve the sluggish reaction kinetics of the ORR, where platinum (Pt) nanoparticles (NPs) have been shown to reach the highest ORR activity, thus serving as a benchmark for the development of new catalysts.^3^ Since platinum is scarce and expensive, various approaches have been established to reduce the required amount of Pt while maintaining high ORR activity.^4,5^ This makes the activity normalized to the mass of active Pt material used (mass activity, MA) a valuable descriptor. One approach is to enhance the catalytic properties by optimizing the catalyst surface structure. The size and shape of NPs play a central role here, and they have been and are still studied extensively.^6^ Interestingly, the activity increase toward the ORR via surface structure modifications of electrocatalysts has been associated with the introduction of catalytically active surface defects, among others.

Calle-Vallejo et al. showed that surface concavities, i.e., hollow or coalescing NPs, have a beneficial effect on the ORR activity for Pt due to an optimized geometric surface, improving the binding energy of the reaction intermediates during ORR.^7,8^ These surface structure considerations were further experimentally proven by various studies.^9,10^ Another vital approach to boost the MA is to alloy Pt NPs with early (e.g., Sc, Y)^11^ or late (e.g., Co, Ni, Cu)^12^ transition-metal elements or lanthanides (e.g., Tb, Pr, Gd),^13,14^ which modifies the electronic properties of the electrocatalyst surface by a combination of so-called strain and ligand effects. While strain effects originate from a mismatch between interatomic distances at the surface and in the bulk of a catalyst, ligand effects stem from the arrangement of Pt atoms with dissimilar neighboring atoms. Both affect the adsorption properties of reaction intermediates, leading to an enhanced electron transfer and, therefore, a higher ORR MA. Transition metals are of particular interest due to their abundance and low cost. For example, alloyed NPs of Pt with Ni or Co have already proven their good performance in activity measurements using the rotating disk electrode (RDE), as well as in fuel cell applications.^15–18^ Alloying Pt with Cu holds great potential as a high-performance ORR catalyst since PtCu alloys were determined to have one of the highest MA increases relative to pure platinum due to an optimized strain effect induced by the right size mismatch between Pt and Cu atoms.^19,20^

In most cases, the synthesis of active nanostructured electrocatalysts relies on conventional bottom-up techniques, which either suffer from complex and time-consuming multistep synthetic procedures or require the use of surfactants or structure-capping agents to control the NP size and shape.^21,22^ Such surfactants block catalytically active sites, which makes additional measures for efficient removal inevitable to maintain the elevated activity.^23,24^ Therefore, we present an alternative method to synthesize PtCu electrocatalyst NPs with record-breaking activity for the ORR based on a surfactant-free, single-step, and scalable top-down approach, which we call electrochemical erosion. The technique is based on the observations of Bredig and Haber over a century ago, in which “metal dust” was formed when an electric potential was applied to metal wires.^25^ In recent years, the groups of Koper^26–29^ and Li^30–32^ further explored this phenomenon and established strategies to produce nanostructures of various metals, including Rh, Bi, Sn, Pb, Au, Ag, Pt, Ni, and Cu, among others, as well as some of their alloys. Although the exact mechanism of nanostructure formation via this technique is still not fully elucidated, individual hypotheses were developed by several groups, ranging from the formation of metal anions,^26^ alkali cation-stabilized metal hydrides,^33^ over ternary metal-hydride complexes,^28^ to electrolyte cation intercalation into the host metal.^34^ This is why more experimental and theoretical work on novel material systems needs to be conducted to get additional insights into the working mechanisms behind the synthesis approach of electrochemical erosion. By applying rather extreme alternating anodic–cathodic potential variations to metal wires immersed in aqueous electrolytes to induce electrochemical erosion, our group successfully produced Pt,^10,35^ Pt_*x*_Pr,^14^ and Pd NPs,^36^ as well as Ti_9_O_17_ nanowires.^37^ Fichtner et al. demonstrated that electrochemical erosion leads to Pt NPs with a large number of surface defects, i.e., surface concavities, enabling an unprecedented ORR activity.^10^ Furthermore, they introduced a strategy to synthesize highly active Pt_*x*_Pr NPs via electrochemical erosion of a bulk Pt_5_Pr disk electrode as a host material.^14^

In this work, PtCu NPs supported on Vulcan carbon with a remarkable activity for the ORR have been produced for the first time using the electrochemical erosion approach. In contrast to previous studies, no bulk alloy wires or alloy disk electrodes were used. Instead, Pt wires immersed in different concentrations of CuSO_4_ mixed into the standard synthesis solution were employed to form alloyed PtCu NPs. The formation of alloyed PtCu NPs with complex grain structures and a high degree of surface defects was confirmed by a combination of high-resolution transmission electron microscopy (HR-TEM), scanning TEM (STEM), energy-dispersive X-ray spectroscopy (EDX), X-ray diffraction (XRD), and X-ray photoelectron spectroscopy (XPS) analysis. Further electrochemical testing revealed an ORR activity superior to that of other Pt and PtCu catalysts in the literature. Our PtCu/C catalysts demonstrate a more than 2-fold increase in MA compared to the revolutionary PtCu core–shell NPs reported by Strasser et al.^20^ and previously reported Pt-based catalysts synthesized via electrochemical erosion.^10,14^

## Results and Discussion

For the synthesis of supported PtCu NPs, a 1 M KOH solution was prepared containing different amounts of CuSO_4_ (0.4 mM to 0.9 mM) as the source of Cu for the alloying process. Vulcan carbon was added as a support material to the synthesis solution after a successful dispersion of KOH and CuSO_4_. Subsequently, two Pt wires were immersed, and an alternating sinusoidal voltage with a frequency of 200 Hz and an amplitude ranging from 10 to 25 V was applied between them, as shown in Figure 1. The erosion process started immediately, and the formed NPs were attached to the carbon support material. The synthesis solution was continuously subjected to ultrasonication and mechanical stirring for better dispersion of the carbon support and a more homogeneous distribution of the eroded NPs. After filtration, washing, and drying, the PtCu/C catalyst powder was obtained. The CuSO_4_ concentration and the applied potential amplitude during the electrochemical erosion were varied from 0.4 mM to 0.9 mM (at a fixed amplitude of 15 V at 200 Hz) and 10 V–25 V (at a fixed CuSO_4_ concentration of 0.54 mM), respectively, as these parameters most likely influence the synthesis of highly active PtCu NPs. Figure S1 shows the atomic concentration of Pt and Cu for the different samples studied in this work, based on inductively coupled plasma mass spectrometry (ICP-MS). All investigated samples showed a Cu atomic ratio between 30% and 50%. Interestingly, an increase in the CuSO_4_ concentration does not lead to a steady increase in the atomic ratio of Cu in the PtCu NPs according to the ICP-MS findings. Instead, a peak can be observed at a concentration of about 0.54 mM, yielding an approximate 1:1 atomic ratio of Pt and Cu. Varying the synthesis potential amplitude leads to only minor changes in the atomic composition of the PtCu NPs, which appears to be rather stochastic. This observation is in agreement with the findings of Garlyyev et al., who reported larger particle sizes with increasing potential amplitude for pure Pt NPs,^35^ which consequently might not directly influence the atomic ratio between Pt and Cu. However, it must be noted that there is no common understanding of the electrochemical erosion mechanism as of now, and operando or in situ studies have not been conducted yet due to the harsh conditions. Hence, a precise explanation for the observed alloying and atomic ratios of Cu and Pt cannot be given. Nevertheless, a higher atomic ratio of Cu would not only save scarce and expensive Pt, but it might also induce more strain in the PtCu NPs, potentially yielding a higher ORR activity due to a weaker oxygen binding energy. Therefore, the PtCu/C catalyst sample synthesized in 1 M KOH with 0.54 mM CuSO_4_ and an applied potential amplitude of 15 V with an approximate 1:1 atomic ratio of Pt and Cu will be the focus in the following, while a detailed summary of the obtained results of all synthesized catalysts can be found in the Supporting Information.

**Figure 1:**
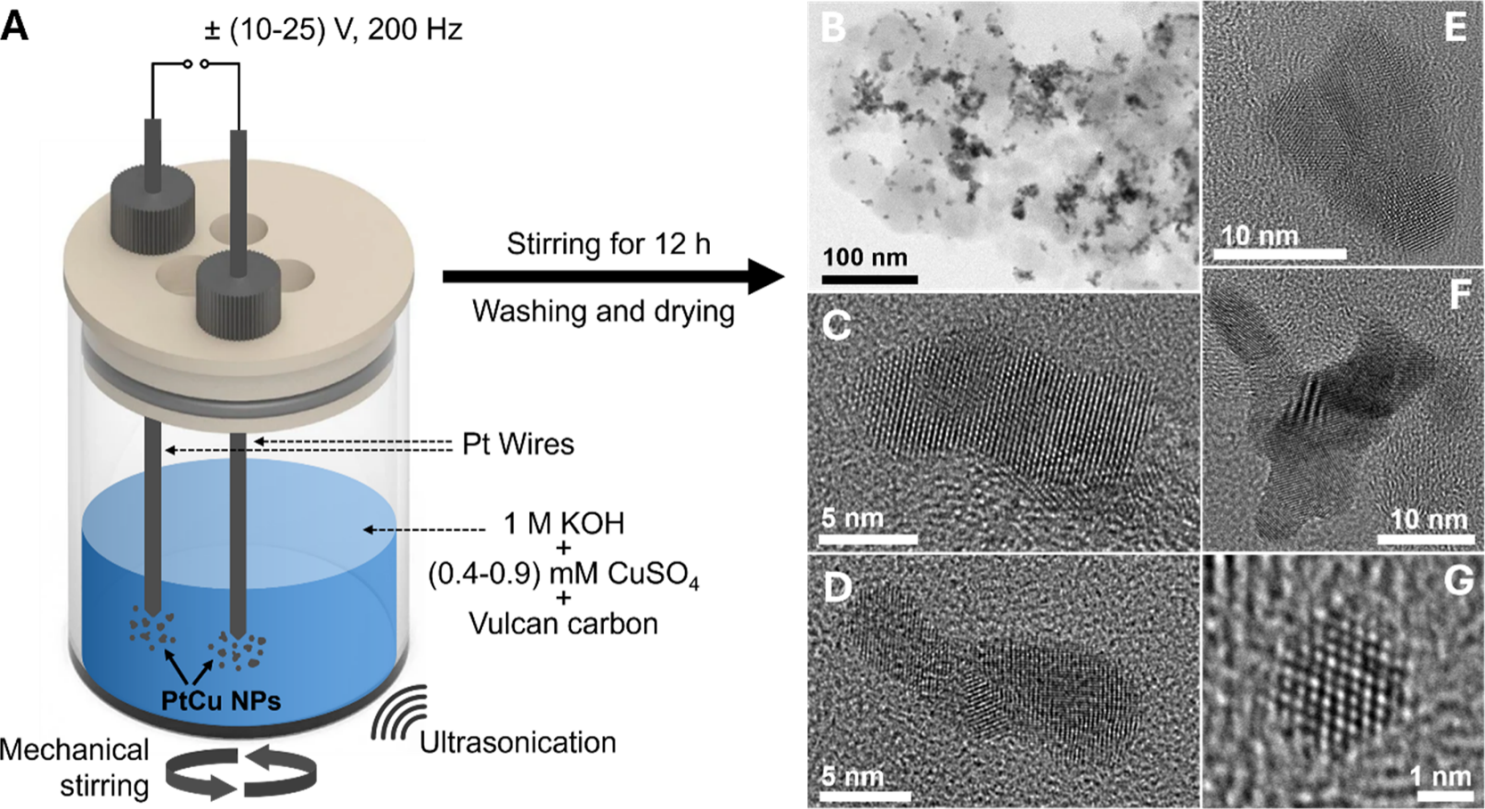
(A) Schematic illustration of the synthesis of PtCu NPs supported on Vulcan carbon via electrochemical erosion. An alternating potential signal (10 V–25 V amplitude, 200 Hz frequency) between two Pt wires in a suspension of Vulcan carbon, 1 M KOH, and CuSO_4_ (0.4 mM–0.9 mM) leads to the formation of PtCu NPs. (B) Overview TEM image shows the distribution of NPs on the carbon support. (C–F) HR-TEM images of selected single PtCu NPs, synthesized in 0.54 mM CuSO_4_ with a 15 V potential amplitude, with irregular shapes and surface concavities after electrochemical cycling. (G) HR-TEM image of a ∼2 nm-sized spherical nanoparticle (0.54 mM CuSO_4_ and 15 V amplitude) after electrochemical cycling.

In the following, we present a detailed characterization of the PtCu/C catalyst synthesized in 1 M KOH with 0.54 mM CuSO_4_ using an applied potential amplitude of 15 V and a frequency of 200 Hz via TEM, XPS, and XRD, followed by its detailed electrochemical evaluation. Since catalyst NPs are immediately subjected to transformational changes during electrochemical operation,^38–40^ we paid special attention to the characterization results of the PtCu NPs after extended electrochemical cycling. We employed a common accelerated stress test (AST) protocol by cycling the catalyst with an accelerated scan rate (100 mV s^–1^) for 1000 cycles under ORR conditions,^14,41^ which is shown in Figure S2. This way, valuable information about the NP shape, size, composition, and structure closer to the actual operational state during electrochemistry can be obtained. In addition, sufficient characterization of the pristine catalyst before any electrochemical treatments is provided to track potential transformations of the PtCu/C catalyst system.

Figure 1B shows a representative TEM image of the synthesized PtCu/C catalyst with a relatively homogeneous distribution of nanoparticles on the carbon support. Selected single NPs after electrochemical cycling through the AST are shown in Figure 1C–G, while Figure S3A–C shows individual pristine NPs. Besides a small contribution of very small spherical particles with diameters between 2 and 4 nm, most particles are larger in size and exhibit rather nonuniform shapes with surface distortion and concavities, as previously observed by electrochemical erosion studies.^10,14^ Most likely, the harsh synthesis conditions drive the Pt to abruptly come off the bulk wire’s surface by applying a large alternating potential (±15 V) with a high frequency (200 Hz), forming irregular particle shapes in contrast to NPs grown via classical wet-chemical bottom-up approaches.^42^ As a result, these nanoparticles contain a high density of surface defects and concave surface sites, shown to enhance the global ORR activity.^8,43^ Therefore, estimating an average size distribution of the synthesized NPs is complicated. Based on the HR-TEM images in Figure 1, the nanostructures vary in size, ranging from ∼5 nm to ∼20 nm. Moreover, the comparison of individual nanostructures in Figures 1 and S3 did not reveal significant changes in the size or shape of the NPs upon AST cycling. Fast Fourier transformation (FFT) analysis of HR-TEM images of representative NPs before and after AST revealed a crystallographic structure response close to that of the Pt *fcc* lattice structure. Small deviations of the estimated lattice spacings from the theoretical Pt values could stem from lattice strain either through surface distortions by the electrochemical erosion approach itself,^10,37^ or through alloying and subsequent acid leaching-induced dealloying during the conducted AST,^20^ which introduces lattice strain through the rearrangement of surface Pt atoms. Additional selected area electron diffraction (SAED) and FFT measurements before and after AST are shown in Figure S4A–E, respectively. These confirm the previous statements and suggest alloy formation through a mixed solid solution, excluding the formation of intermetallic compounds, as elaborated in detail in the Supporting Information. In summary, the structural analysis via TEM did not reveal significant changes between before and after AST except for a small alteration of the lattice spacings, which could stem from acid-leaching-induced dealloying. As a result, the synthesized PtCu NPs can be assumed to exhibit good structural stability upon electrochemical cycling.

Analyzing the nonspherical and irregular-shaped NPs in Figure 1C–E in more detail, different crystalline domains or lattice orientations within a single particle become visible. We conducted an in-depth investigation via FFT of two exemplary nanoparticles to gain more information about the complex shape and structure of the electrochemical erosion-derived PtCu/C catalyst. Figure 2A shows the HR-TEM image of the nanoparticle depicted in Figure 1E with different crystalline domains marked in purple. Exemplary FFT reflection patterns are provided in Figure 2B,C for areas 1 and 2a, respectively. The areas of the FFT evaluation, together with the FFT results of other areas, can be seen in Figure S5 in the Supporting Information. Through careful evaluation of these patterns, we were able to estimate five distinct crystalline domains within this exemplary nanoparticle, with some containing additional subdomains, which revealed slightly different FFT values. In detail, various lattice planes, i.e., (111), (200), (220) for area 1, and (220), (242), (602) for area 2a, were identified, assuming a Pt *fcc* crystal structure with symmetry space group *Fm*3̅*m*, aligning with the XRD results, which will be discussed later. As a result, the zone axes were determined as [011] and [1̅13], respectively. For all other NP domains, the FFT results could not provide sufficient information to accurately define the zone axes due to a lack of lattice symmetry, i.e., periodicity, in these areas. This could be a consequence of surface defects, as previously observed for Pt nanoparticles in a similar electrochemical erosion study,^10^ or of alloying and subsequent dealloying through electrochemical cycling.^20^ Nevertheless, we determined multiple lattice spacings for the sketched areas in Figure S5. The nanoparticle areas, their corresponding FFTs, and the estimated lattice spacings are listed in Table S1 in the Supporting Information. The obtained values deviate by up to ∼20 pm from the literature values given in Table S2 for Pt and Cu, consistent with the discussion provided by Figure S3 regarding the FFT. These deviations could result from local defects and strains, which alter the determined *d*-spacing values due to local lattice contraction or expansion. Consequently, variations in the degree of local strain between the crystalline domains could explain the relatively large deviation of the assessed *d*-spacing values from the reference data for certain nanoparticle areas. Since the *d*-spacings, and therefore, the lattice parameters of Cu (∼3.615 Å) and Pt (∼3.923 Å) are very similar, it is challenging to assign the resulting values to specific Pt or Cu crystal facets. Nonetheless, the deviations further confirm the high degree of disorder and strain in the PtCu nanoparticles derived by the electrochemical erosion approach. Still, we provided estimates of the lattice orientations at the nanoparticle surface, as illustrated for areas 1, 2a, and 2b in Figure 2A, in Table S1, based on the FFT results. From this data, we deduced the highlighted areas of different crystalline domains in Figure 2A. Most of these areas are estimated to be linked to Pt (111) or Pt (220) lattices, which, however, cannot be assigned accurately. Nevertheless, different crystalline domains, i.e., grains and corresponding grain boundaries, could be identified, proving the complex morphology and structure of the PtCu nanoparticles derived by the electrochemical erosion approach. Therefore, we conclude that a large quantity of the PtCu nanoparticles is composed of multiple crystalline domains, ∼3–6 nm in size, interconnected by grain boundaries. Interestingly, such features have been proposed to enhance the ORR performance by optimizing the reaction intermediates’ binding energies.^10,43^

**Figure 2:**
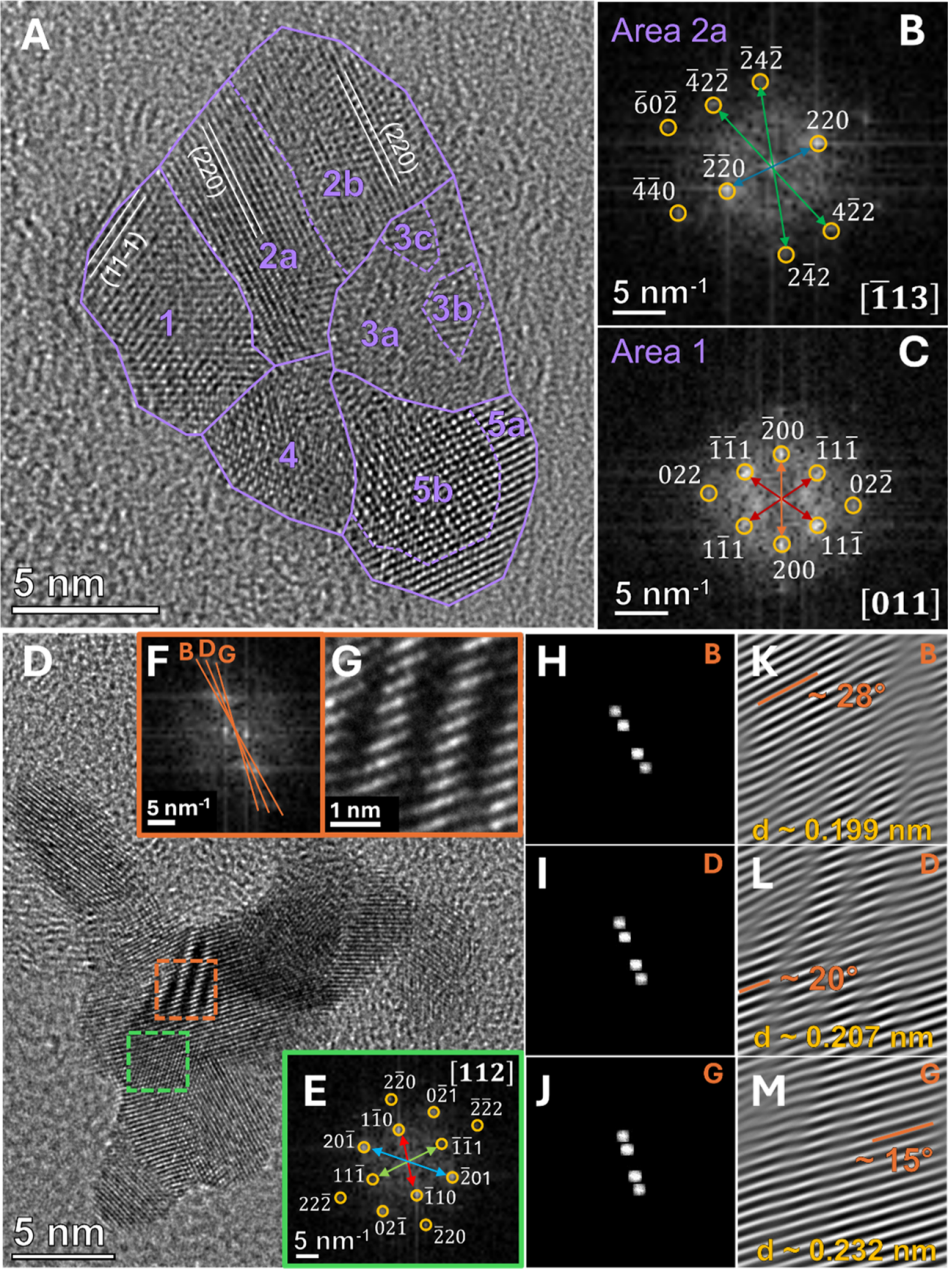
(A) HR-TEM image of an individual nanoparticle, synthesized in 0.54 mM CuSO_4_ with a potential amplitude and frequency of 15 V and 200 Hz, respectively, with highlighted crystalline domains as estimated by FFT analysis. (B,C) FFT reflection patterns of area 1 and 2a of the particle shown in (A). (D) HR-TEM image of an individual nanoparticle, synthesized in 0.54 mM CuSO_4_ with a 15 V potential amplitude. The dashed green and orange rectangles show selected areas for FFT evaluation. (E) FFT reflection pattern of the area marked in green in (D). (F,G) FFT reflection pattern and HR-TEM magnification, respectively, of the area marked in orange in (D). (H–J) corresponding FFT masks of the three planes B, D, and G, identified in (F), respectively. (K–M) inverse FFT patterns of the three planes B, D, and G, respectively, showing the corresponding *d*-spacings and plane angles.

Another exemplary nanostructure with a complex structure is depicted in Figure 2D. All corresponding FFT areas, reflection patterns, identified lattice spacings, and estimated lattice orientations can be found in detail in Figure S6 and Table S3 in the Supporting Information. Similar to the nanoparticle in Figure 2A, the determined *d*-spacings deviate from the reference data of Pt and Cu. Accordingly, lattice orientations of either Pt or Cu, corresponding to Pt(200), Pt(111), or Cu(111), were detected, revealing different crystalline domains within the nanoparticle. Interestingly, two areas, indicated by the dashed green and orange rectangles, revealed distinct FFT responses. The green rectangle, corresponding to the FFT reflection in Figure 2E, revealed an FFT response matching the trigonal CuPt phase, as apparent by the 3-fold rotational symmetry and the presence of forbidden reflections, which are not allowed for pure Cu or Pt. The zone axis was determined to be close to [112]. It implies that Pt and Cu atoms are arranged alternatingly in (111) atomic planes within one parent *fcc* lattice, instead of forming a mixed solid-solution alloy, as observed for the other nanostructures discussed above. The orange rectangle, corresponding to the FFT pattern in Figure 2F, revealed a so-called Moiré pattern, which is visible in the magnified HR-TEM image in Figure 2G. This pattern is a result of interference through the superposition of three lattices with slightly different lattice spacings, rotations, and translations. These are shown in the corresponding FFT masks and inverse FFT patterns in Figure 2H–M, respectively. The three different planes, marked as B, D, and G, exhibit *d*-spacings of ∼0.199 nm, ∼0.207 nm, and ∼0.232 nm, respectively. Their rotations were determined to be ∼28°, ∼20°, and ∼15°, respectively. Moiré patterns are typically only observed in two-dimensional materials, such as thin films,^44^ which makes the formation of Moiré superlattice structures in our three-dimensional nanoparticles a remarkable finding. Two-dimensional Moiré superlattice structures have shown promising activity and stability enhancements in photo- and electrocatalytic applications compared to conventional alternatives.^44,45^ Interestingly, they are often correlated with an altered electronic environment, e.g., the density of states, and the introduction of lattice strain, facilitating efficient electron transfer by lowering kinetic barriers.^44–46^ Although the relative abundance of Moiré patterns in the PtCu nanoparticles may be rather small, they are likely to contribute to an enhanced ORR activity.^45,46^ However, an accurate estimation of its impact on the electrocatalytic performance would require computational support. Unlike in Figure 2A, no individual grains were marked for the nanoparticle in Figure 2D due to the increased complexity in the determination of the apparent structures, such as the trigonal CuPt phase and the Moiré pattern. In conclusion, HR-TEM and FFT analyses revealed that the nanoparticles are composed of multiple grains, separated by grain boundaries and defects. In addition, distinct crystal responses of a trigonal CuPt phase and a Moiré superlattice structure pattern were identified, underlining the complexity of the investigated nanoparticles, as demonstrated by the in-depth characterization in Figure 2. These factors potentially influence the ORR activity besides the alloying between Pt and Cu.

To confirm the presence of Cu in the synthesized NPs, STEM-EDX elemental analysis of representative single NPs was carried out before and after conducting the AST. Figure S7 in the Supporting Information shows the STEM-EDX elemental maps of two individual NPs before electrochemical testing with a homogeneous distribution of Pt and Cu. Based on the EDX analysis, the average elemental ratio between Pt and Cu was calculated to be ∼52:48, which precisely coincides with the results from ICP-MS (∼51:49). More important is the question of whether the PtCu NPs remain stable during electrochemical cycling (which will be shown later), as Pt alloys with less noble metals tend to dealloy under potential bias in acidic environments.^20^ The elemental maps in Figure 3A show a homogeneous distribution of Pt and Cu throughout the entire NP after the AST, supporting the assumption of a stable PtCu alloy formation. A detailed EDX analysis revealed a slightly increased Pt to Cu ratio of ∼59:41 compared to the investigated NPs before cycling (∼52:48). This provides a strong hint toward a partial dealloying of the PtCu NP, most pronounced at the particle surface, as indicated by the Pt, Cu, and combined Pt + Cu elemental maps. The loss of Cu most likely stemmed from acid leaching during electrochemical cycling, resulting in a core–shell structure with a Pt shell and a mixed PtCu core, as proposed in the literature.^20^ Moreover, the intensity profiles in Figure 3B confirm the coexistence of Pt and Cu throughout the entire NP in an approximate 60:40 atomic ratio, taking into account the slightly larger Cu background signal. This further proves the formation of PtCu alloy NPs with a core–shell structure, as observed by Strasser et al.^20^ Interestingly, the EDX maps reveal a uniform distribution of oxygen, suggesting the presence of oxidized Pt or Cu species at the nanoparticle surface, as well as an oxidized carbon support surface. The first could stem from the harsh synthesis conditions, switching continuously between strongly oxidizing and reducing potentials. The latter can be attributed to the surface modification of Vulcan carbon with H_2_O_2_ to increase the hydrophilicity during synthesis. This introduces oxygen functionalities at the carbon surface to improve wettability^47^ ^,^ as described in more detail in the experimental section.

**Figure 3:**
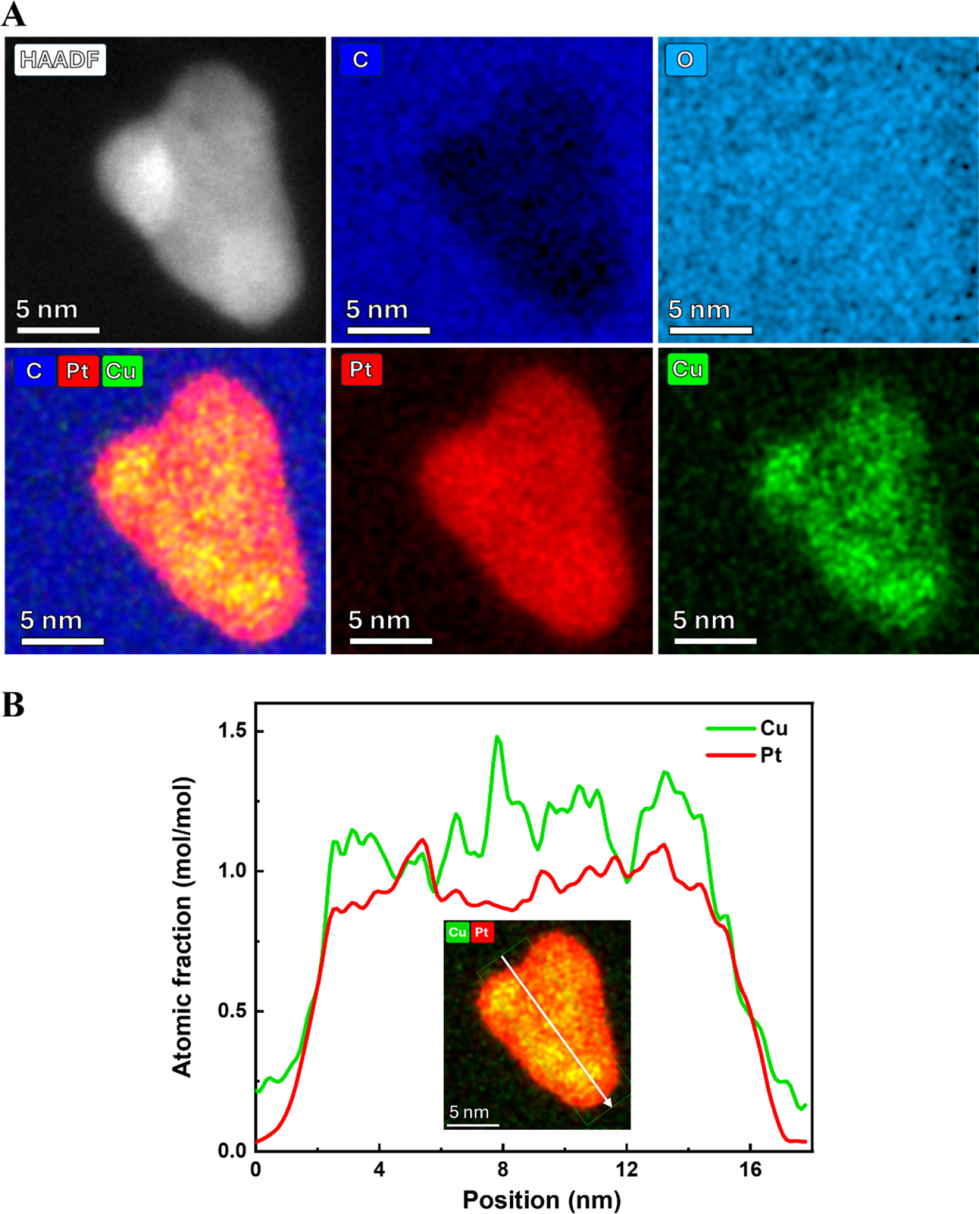
(A) HR-STEM-high-angle annular dark field (HR-STEM-HAADF) image and corresponding EDX elemental maps of a single PtCu NP synthesized in 1 M KOH and 0.54 mM CuSO_4_ with 15 V potential amplitude for Pt–L, Cu–L, C–K, and O–K, as well as combined Pt, Cu, and C after electrochemical cycling. STEM-EDX elemental maps of the PtCu/C sample before electrochemical cycling can be found in the Supporting Information. (B) Corresponding Pt and Cu intensity profiles of the nanoparticle in (A), indicated by the white arrow.

XPS analysis gives further insights into the material’s surface composition and oxidation states. The fitted Pt 4f spectra of the PtCu/C catalyst, synthesized in 1 M KOH and 0.54 mM CuSO_4_ with a potential amplitude of 15 V, are shown in Figure 4A before and after electrochemical cycling. Both fitted spectra propose the presence of mostly metallic Pt and minor contributions from oxidized species (Pt^4+^ and Pt^2+^) in an approximate 3:1 ratio. The fitted Cu 2p_3/2_ spectra, displayed in Figure 4B, suggest the presence of metallic and oxidized Cu (Cu^2+^) with the typical satellite peaks characteristic of the Cu^2+^ state. Interestingly, all spectra indicate an alloy formation between Pt and Cu due to the slightly shifted peak positions of the metallic Pt (∼70.8 eV) and Cu (∼931.7 eV) signals to lower binding energies compared to pure Pt (∼71.2 eV) and Cu (∼932.3 eV).^48–50^ The fitted doublet peaks in the Pt 4f spectra were deconvoluted into two separate peak pairs to emphasize the modified electronic environment by forming a mixed solid-solution PtCu alloy, as previously reported.^49^ The binding between electrons and (sub) surface Pt atoms of the PtCu nanoparticles is a continuous mixture of Pt–Pt and Pt–Cu binding energy states, as recorded by the XPS spectra and visualized by peak pair splitting into Pt^0^ and Pt–Cu. Furthermore, two peaks at ∼937 eV and ∼935 eV have been assigned to Cu–F due to interaction with the Nafion ionomer (part of the catalyst ink used for the XPS measurement) and Cu–OH, respectively. The latter could stem from the interaction of the Cu^2+^ from the dissolved CuSO_4_ and the OH^–^ from the dissolved KOH during the electrochemical erosion process. After the AST, the Cu–F, Cu–OH, and satellite peaks disappeared. Additionally, the ratio between metallic and oxidized Cu increased from ∼2:3 to ∼2:1, indicating a decrease in oxidized Cu on the surface of the PtCu/C catalyst. The ratio between Cu and Pt on the surface of the PtCu/C catalyst decreased from ∼2:3 before to ∼1:6 after the AST. Therefore, our XPS analysis provides strong evidence for Cu leaching during electrochemical cycling, leaving behind NPs with a Pt-rich shell and a mixed PtCu core, as proposed by the STEM-EDX results (see Figure 3). This should prove beneficial for the ORR performance due to the induced lattice strain.^19,20^ The corresponding fitted XPS spectra of the C 1s and O 1s core-level regions and their description can be found in the Supporting Information (Figure S8), as well as the complete spectral range of Cu 2p core-level regions (Figure S9) and an overview table of the position of the identified peaks (Table S4).

**Figure 4:**
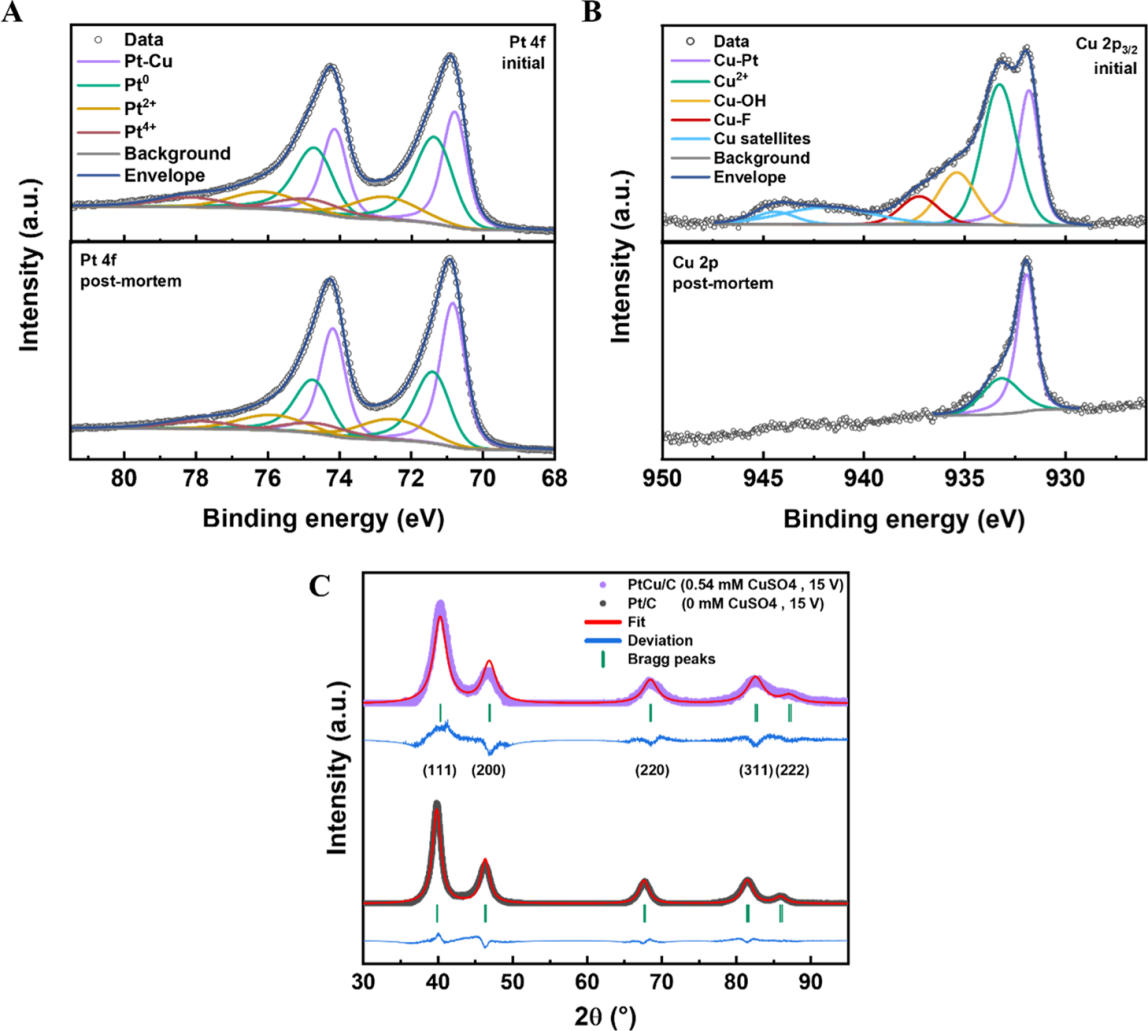
Fitted XPS spectra of the (A) Pt 4f and (B) Cu 2p_3/2_ core-level regions of the PtCu/C catalyst sample synthesized in 1 M KOH and 0.54 mM CuSO_4_ with a 15 V potential amplitude and 200 Hz frequency before and after electrochemical cycling. (C) Background-subtracted powder XRD patterns of PtCu/C and reference Pt/C before electrochemical characterization. Rietveld refinement fits are shown as the red curves. Blue curves visualize the deviation between raw data and fitting. Green tickmarks illustrate the identified Bragg peaks.

XRD analysis of PtCu/C directly after the synthesis in 1 M KOH and 0.54 mM CuSO_4_ with a 15 V potential amplitude is shown in Figure 4C. It reveals an *fcc* structure with peak positions close to that of pure Pt (PDF card no. 04-0802), matching the (111), (200), (220), (311), and (222) reflections. However, all diffraction peaks of the PtCu sample were shifted to higher 2θ values, indicating a contraction of the Pt lattice parameter upon alloying with Cu and confirming the successful formation of a PtCu alloy.^51,52^ Rietveld refinement, using the *Fm*3̅*m* phase and adopting isotropic peak broadening, confirms the phase purity of an *fcc* lattice structure close to that of Pt. The lattice parameter of PtCu/C was determined as ∼3.87 Å, significantly smaller than that of pure Pt (∼3.92 Å), which is expected since the lattice parameter of Cu is much smaller (∼3.62 Å). Therefore, the XRD analysis suggests the formation of a mixed solid-solution alloy of Pt and Cu with a lattice parameter between that of Pt and Cu, which is in agreement with the lattice distortions found by FFT analysis in Figures 2, S3 and S4. From this, the lattice contraction can be calculated, which amounts to approximately 1.2% for the PtCu/C catalyst. Moreover, the XRD results are consistent with the lattice spacing variations obtained from FFT analysis of the PtCu/C catalyst relative to pure Pt, confirming that lattice distortion is present in the crystal lattice. To validate the origin of the lattice contraction, a Pt/C catalyst was produced using the same parameters without the addition of CuSO_4_ (gray curve), whose peak positions align well with those of the pure Pt reference (PDF card number 04-0802). The corresponding lattice parameter almost coincides with that of pure Pt, revealing only a small lattice contraction of ∼0.2%. The reason for a contraction value unequal to zero could be the presence of irregularly shaped nanoparticles with surface defects and distortions, as observed by Fichtner et al.^10^ The large difference in lattice parameter for PtCu/C indicates that the 2θ angle shift primarily stems from alloying instead of the relatively harsh synthesis conditions. The small peak at ∼35° corresponds to the strongest (111̅) reflection of monoclinic CuO, also known as mineral tenorite (PDF card number 45-937), which could be a residual of the synthesis by employing KOH and CuSO_4_.

XRD patterns, corresponding Rietveld refinements, and deduced parameters of the PtCu catalysts fabricated under different CuSO_4_ concentrations and applied alternating potential amplitudes can be found in the Supporting Information (see Figures S10, S11, and S12 and Table S5) with a detailed description of the results. All samples exhibit distinct 2θ peak shifts compared to pure Pt and could be modeled using an *fcc* lattice structure in the *Fm*3̅*m* space group. Besides the different trends observed for modifying the CuSO4 concentration and alternating potential amplitude, all PtCu/C catalysts exhibit a smaller lattice parameter than the reference Pt/C catalyst and Pt from the database, validating the success of our one-pot synthesis approach for preparing alloyed PtCu NPs. Furthermore, the XRD results comprise the average crystallite size of the synthesized catalysts, which all lie between 2.6 and 2.9 nm, as illustrated in Figure S12C,D. No clear trend in the average crystallite size was observed upon varying the CuSO_4_ concentration or synthesis potential amplitude. As a commonly used parameter for roughly estimating the nanoparticle size, this information provides valuable insight into the atomic-scale structure of the PtCu NPs. For instance, the PtCu/C catalyst synthesized in 0.54 mM CuSO_4_ and with an amplitude of 15 V exhibits an average crystallite size of (2.9 ± 1.1) nm, considerably smaller than the individual NPs observed by HR-TEM. The data derived from Figure 2 could provide an explanation for this apparent discrepancy. In-depth analysis revealed complex, nonuniform shapes of the PtCu NPs, which appear to be composed of multiple small lattice domains separated by grain boundaries with sizes ranging from ∼3 nm to ∼6 nm. Therefore, the assessed average crystalline size of approximately 3 nm, determined via Rietveld refinement, matches the size of the identified crystalline domains reasonably well, in contrast to the overall nanoparticle dimension.

As mentioned earlier, various mechanisms for the formation of NPs by this top-down approach have been proposed so far. However, still no clear consensus has been reached. By fabricating alloyed PtCu NPs, in which the two metals coexist in distinct physical phases, this work provides new insight into the underlying formation mechanism. In the following, a hypothesis for the formation of PtCu alloy NPs is presented, based on the observations and characterizations discussed above: numerous studies were conducted to explain noble metal dissolution during electrochemical cycling.^53–55^ Most likely, Pt is oxidized during the anodic scan and subsequently reduced during the cathodic scan. The latter process results in the partial dissolution of Pt species into the electrolyte solution.^53,54^ The dissolved Pt species are typically reported to be of cationic nature,^53,56^ even though some reports about anionic species exist.^26,38,54^ When reaching more cathodic potentials, redeposition of the previously dissolved Pt species onto the bulk Pt wire could take place.^57^ Simultaneously, Cu from the electrolyte, present as Cu^2+^ complexed by hydroxo ligands as [Cu^2+^(OH)_4_(H_2_O)_2_]^2–^ at pH 14, could be deposited onto the bulk Pt wire’s surface.^58^ The redeposition of dissolved Pt species together with Cu could result in the formation of homogeneous PtCu alloy layers on the bulk Pt wire’s surface.^58^ When returning to anodic potentials, deposited Cu and Pt would then be oxidized and dissolved again, completing the cycle. This process of alternating dissolution and redeposition could repeat itself until larger local PtCu domains break out of the bulk Pt wire’s surface, yielding the PtCu NPs, which subsequently attach to the carbon support. The formation of larger domains locally on the bulk Pt wire’s surface could be due to the fact that dissolution might preferably start at local surface defects or concavities.^36,57^ Therefore, continuous dissolution and redeposition would most probably not result in homogeneous layers of PtCu, but rather in a rough surface structure with larger local PtCu domains, promoting the break out of PtCu NPs upon increasing mechanical instability.^59^ Applying high alternating potentials (10 V–25 V amplitude) with a very high frequency (200 Hz), i.e., a very fast scan rate, could accelerate the dissolution and successful redeposition and, therefore, the formation of PtCu NPs.^60^ Furthermore, the mechanical instability of the inhomogeneously grown PtCu domains on the bulk Pt wire’s surface could be further amplified by the strong hydrogen or oxygen evolution occurring at these potentials, causing “electrochemical erosion’’ to accelerate the detachment of PtCu NPs from the bulk Pt wire’s surface. This could not only explain alloy formation between Pt from the bulk host electrode and Cu from the electrolyte solution but also the nonuniform NP shapes observed in Figure 1.

As elucidated earlier, incorporating transition metals like Cu, with smaller atomic radii, into the Pt lattice decreases the average interatomic Pt bond lengths, resulting in a lattice contraction that can be considered as local strain. The corresponding downward shift of the Pt d-band center weakens the adsorption strength of the ORR intermediates, thereby enhancing the overall ORR activity.^4,20^ Therefore, the ORR activities of the fabricated PtCu catalysts were evaluated in acidic media via cyclic voltammetry utilizing the RDE technique. Figure 5A shows a typical cyclic voltammogram (CV) in Ar-saturated 0.1 M HClO_4_ for the PtCu/C catalyst synthesized in 1 M KOH and 0.54 mM CuSO_4_ with 15 V potential amplitude before and after AST. Characteristic peaks in the hydrogen underpotential deposition (H_UPD_) region (0.05 V–0.35 V vs the reversible hydrogen electrode (RHE)) and surface oxide formation and reduction peaks (0.6 V–1.2 V vs RHE) can be seen, revealing the characteristic features of Pt-based NPs and allowing for the determination of the electrochemically active surface area (ECSA). Changing the CuSO_4_ concentration and the potential amplitude during synthesis only has a minimal influence on the CVs, as presented in Figure S13A,B, respectively. Dividing the ECSA by the Pt mass loadings obtained via ICP-MS (see Table S6) provides the specific surface area (SSA), as displayed in Figure S13C,D. The PtCu/C electrocatalyst synthesized with 0.54 mM CuSO_4_ reached the highest SSA of (47 ± 2) m^2^ g_Pt_^–1^ among the samples of varied concentrations (see Supporting Information for a detailed description of the SSA trends). This value is smaller than that of commonly reported commercial Pt/C catalysts (∼70 m^2^ g_Pt_^–1^),^14,61^ as also indicated by less pronounced H_UPD_ peaks in Figure 5C. The lower H_UPD_ peak current of PtCu/C compared to commercial Pt/C_TKK_ (Tanaka) indicates less hydrogen adsorption and desorption, resulting in a smaller ECSA and SSA. This could be a result of the larger and nonuniform NPs prepared in this study via electrochemical erosion in contrast to commercial Pt/C catalysts.^62^ Additionally, PtCu/C shows a distinct peak shoulder at ∼0.26 V vs RHE (green dotted line) compared to the peaks at ∼0.15 V and ∼0.22 V vs RHE (gray dotted lines) of commercial Pt/C that can be assigned to Pt(110) facets.^63^ The additional peak at ∼0.26 V vs RHE could be explained by hydrogen desorption on defective surfaces, as suggested by Chattot et al.,^64^ which delivers further evidence of the concave defect-rich NP surface of PtCu/C. A detailed summary of all evaluated electrocatalytic measures can be found in Table S7.

**Figure 5:**
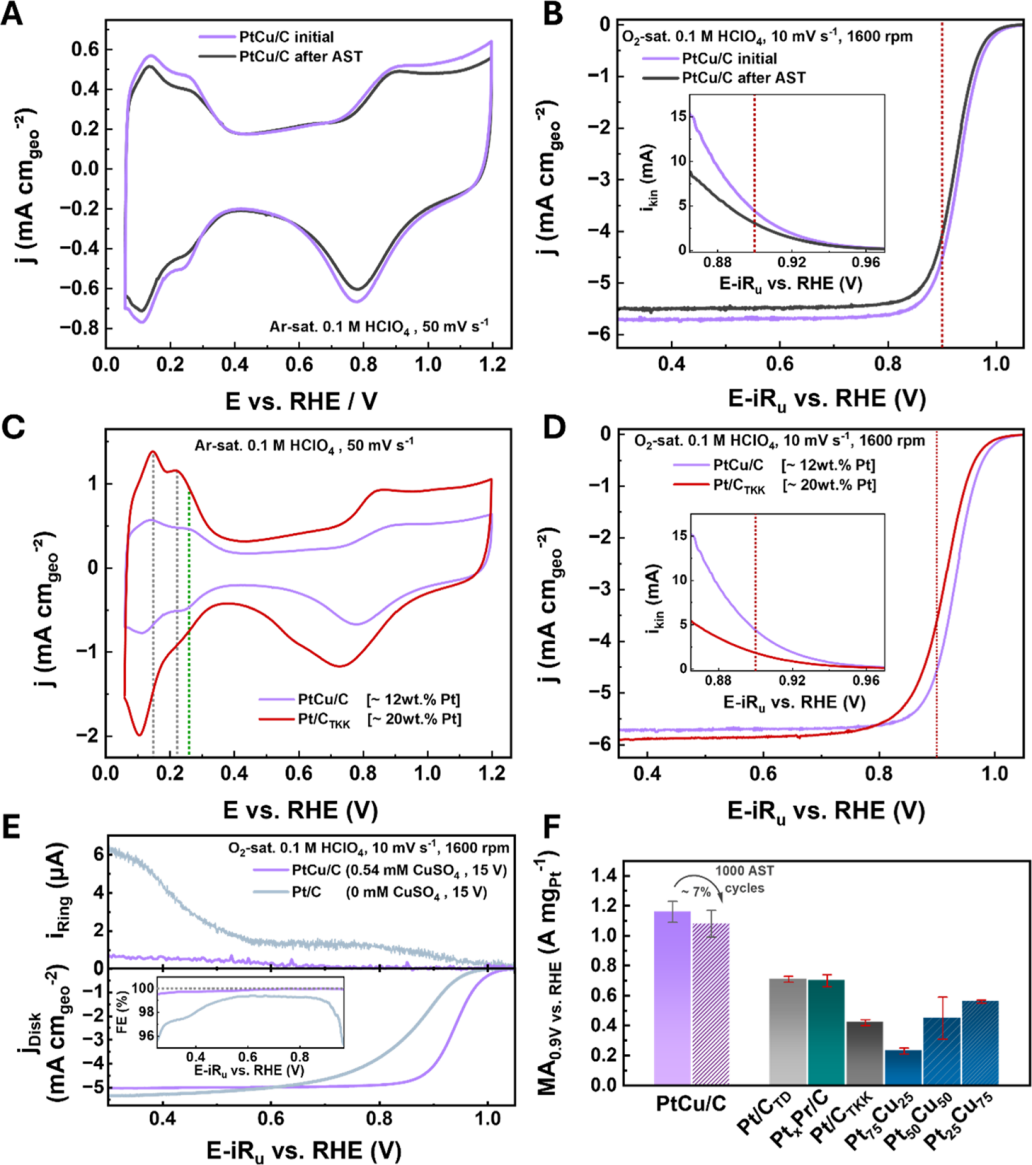
(A) Typical CVs of PtCu/C, synthesized in 1 M KOH and 0.54 mM CuSO_4_ with 15 V potential amplitude and 200 Hz frequency, in Ar-saturated 0.1 M HClO_4_ at 50 mV s^–1^ scan rate. (B) Typical *iR*-corrected anodic polarization curves in O_2_-saturated 0.1 M HClO_4_ at 10 mV s^–1^ scan rate and 1600 rpm rotation speed. (C) Comparison of PtCu/C with commercial Pt/C_TKK_ from Tanaka in Ar-saturated 0.1 M HClO_4_ at 50 mV s^–1^ scan rate. (D) Corresponding ORR polarization curves in O_2_-saturated 0.1 M HClO_4_ at 10 mV s^–1^ scan rate and 1600 rpm. (E) Typical RRDE measurement of PtCu/C and the reference Pt/C catalyst, synthesized without CuSO_4_, showing the disk current density of the ORR polarization curve (bottom) and the corresponding ring current (top). The calculated FEs using the determined collection efficiency of the RRDE setup (*n* ∼ 22%) are shown as an inset in (E). (F) MA overview of several Pt-based electrocatalysts toward the ORR at 0.9 V vs RHE in O_2_-saturated 0.1 M HClO_4_. The PtCu/C catalyst is compared with Pt/C_TD_ and Pt_*x*_Pr/C from refs 10 and 14 respectively, synthesized via electrochemical erosion. Data of commercial Pt/C_TKK_, Pt/C_TD_, and Pt_*x*_Pr/C was adapted with permission from refs 10 and 14 respectively. MAs of PtCu core–shell NPs from Strasser et al. were adapted with permission from ref 20. A more detailed comparison with catalysts from the literature can be found in Table S8 and Figure S15. The insets in (B,D) show the calculated kinetic currents. The vertical red bars mark the potential at which the ORR activities were determined (0.9 V vs RHE).

A typical ORR polarization curve is given in Figure 5B for PtCu/C synthesized in 1 M KOH and 0.54 mM CuSO_4_ with a potential amplitude of 15 V before and after AST. Taking the Pt mass loading and the ECSA, together with the kinetic current displayed in the inset of Figure 5B, we derived a high specific activity (SA) of (2.5 ± 0.2) mA cm^–2^ and a remarkable MA of (1.2 ± 0.1) A mg_Pt_^–1^ at 0.9 V vs RHE. This corresponds to a 4-fold and 3-fold increase in SA and MA, respectively, compared to the commercial Pt/C_TKK_ catalyst, as indicated in Figure 5D, despite its lower SSA, demonstrating the enhancement of the catalyst’s intrinsic activity through strain and ligand effects. Furthermore, it also demonstrates a clear ORR performance enhancement compared to commercially available Pt alloy catalysts from Umicore (∼5-fold MA increase)^40^ or Tanaka (∼3-fold MA increase).^65,66^ Moreover, an almost 2-fold increase in MA was achieved compared to the best-performing Pt-based catalysts prepared by electrochemical erosion so far.^10,14,35^ This proves that our one-step top-down synthesis not only yielded the successful formation of PtCu NPs but also that the alloying between Cu and Pt indeed strongly enhanced the ORR kinetics, boosting the MA. This also means that alloys can not only be obtained successfully by using a bulk alloy electrode as in our previous publication,^14^ but also through the use of inorganic compounds, such as CuSO_4_, mixed into the KOH solution. This could open the door to the fabrication of many more metal alloys employing the electrochemical erosion approach. Additionally, the exceptional ORR activity could be attributed to a synergistic interplay between Cu–Pt alloying, surface coordination through surface defects, such as concavities, and the presence of strain, which may arise from dealloying, grain boundaries, and Moiré structures, as detailed in Figure 2. Furthermore, rotating ring disk electrode (RRDE) experiments (see Figure 5E) were conducted to probe whether harmful H_2_O_2_ was produced that would corrode fuel cell components over time. To this end, the central disk electrode was coated with the catalyst material to mimic O_2_ reduction, while the surrounding rotating ring electrode would electrochemically detect any H_2_O_2_ formation. The absence of such a ring current confirmed a successful four-electron transfer mechanism and revealed a faradaic efficiency of ∼100% toward the full reduction of O_2_ to H_2_O in the entire potential window of the recorded polarization curve. In contrast, for the pure Pt reference catalyst, synthesized identically without CuSO_4_ (see Experimental Section), a higher ring current was observed, leading to an overall lower faradaic efficiency (FE) toward the four-electron ORR. Therefore, the incorporation of Cu during electrochemical erosion not only yields a four-electron ORR activity enhancement but also demonstrates a lower H_2_O_2_/higher H_2_O faradaic efficiency. This way, the electrocatalytic results confirm the successful formation of PtCu alloy nanoparticles with optimized binding energy of the ORR intermediates. Additional Koutecký–Levich analysis proved the number of transferred electrons to be ∼4 over a wide potential range, as displayed in Figure S14, thus endorsing an efficient four-electron reduction of O_2_ to H_2_O on our highly active PtCu/C catalyst. Polarization curves of the PtCu/C catalysts synthesized in 1 M KOH and 200 Hz frequency with different CuSO_4_ concentrations and potential amplitudes are provided in the Supporting Information (Figure S16).

Comparing the MA of the produced PtCu/C catalysts in Figure S17A,B, it becomes apparent that a 0.54 mM CuSO_4_ concentration yields the maximum MA, while changing the amplitude of the applied AC potential does not significantly affect it. According to the Rietveld refinement results, the lattice contraction, i.e., the introduced strain, increases with higher CuSO_4_ concentration used during synthesis. Therefore, we conclude that a CuSO_4_ concentration of 0.54 mM induces an optimal average lattice contraction, i.e., lattice strain, of ∼1.3%, optimizing the binding interaction between Pt surface sites and ORR intermediates for the electrochemical erosion-derived PtCu/C electrocatalysts. Nevertheless, it should be noted that the XRD results reflect averaged values, since an accurate characterization of the lattice strain of individual nanoparticles is challenging. A more detailed discussion is provided in the Supporting Information. Regarding the SA (Figure S17C,D), the fabricated catalysts reach remarkable values of ∼2.5 mA cm^–2^ at 0.9 V vs RHE, independent of the CuSO_4_ concentration. In contrast, the SA of the PtCu/C catalysts derived from different potential amplitudes follows the opposite trend as assessed for the SSA, reaching an SA of up to (3.0 ± 0.6) mA cm^–2^ for a potential amplitude of 25 V. The outstanding activities of the PtCu/C catalysts obtained in this study, compared to other Pt-based electrocatalysts in the literature, as shown in Figure 5F, may be attributed to a synergistic effect of alloy formation and surface defects. Both effects are well-known to enhance the ORR activity and could result in the superior ORR activity of the PtCu/C electrocatalyst fabricated in this study.

To test the stability of the PtCu/C electrocatalysts, ASTs were conducted, as mentioned before, by running 1000 cycles in O_2_-saturated 0.1 M HClO_4_ at a scan rate of 100 mV s^–1^ and a rotation speed of 1600 rpm. A representative CV and polarization curve after a performed AST are displayed in Figure 5A,B (dark gray) for the sample synthesized in 0.54 mM CuSO_4_ with a 15 V potential amplitude, showing only minor changes. Overall, the ASTs for all synthesized PtCu/C catalysts revealed <10% losses in MA, SA, and SSA, as shown in detail in Figures S13 and S17. Compared to the results for commercial Pt/C_TKK_ from Tanaka in Figure S18, it becomes clear that our synthesized PtCu/C catalyst not only outperforms its commercial counterpart, but also proves to be more stable upon 1000 AST cycles due to a substantially smaller relative loss in both MA and SA. Thus, our prepared PtCu/C catalysts exhibit excellent electrocatalytic stability. However, further tests need to be implemented under experimental conditions closer to the real application in fuel cells, i.e., in membrane electrode assemblies in a fuel cell setup or at elevated temperatures, to gain more reliable stability data, which is, however, not the scope of this work (see Supporting Information for more information).^67^

## Conclusion

In this work, a simple one-step top-down approach was employed to produce PtCu/C electrocatalysts by adding CuSO_4_ salt to the synthesis electrolyte solution, thereby circumventing complex multistep procedures and the use of surfactants or capping agents. HR-TEM imaging with EDX elemental mapping proved the formation of PtCu NPs with a pronounced nonuniform size and shape character. XPS and XRD analysis further confirmed the successful alloying of Pt with Cu. Using a CuSO_4_ concentration of 0.54 mM and a potential amplitude of 15 V during the electrochemical erosion synthesis yielded PtCu nanoparticles with an atomic ratio of 1:1 between Pt and Cu. The voltammetric characterization with an RDE revealed superior ORR performance, with a mass activity of ∼1.2 A mg_Pt_^–1^, a specific activity of ∼2.5 mA cm^–2^ at 0.9 V vs RHE, and excellent stability. This corresponds to a ∼3-fold and ∼4-fold improvement compared to the commercial Pt/C catalyst, respectively, and an almost 2-fold increase in mass activity compared to previously reported Pt-based catalysts synthesized via the electrochemical erosion method. Based on this investigation, our PtCu/C holds great potential as a promising ORR electrocatalyst for the application in fuel cell membrane electrode assemblies, particularly considering the simple, scalable, and environmentally friendly fabrication of the one-step top-down approach presented herein.

## Experimental Section

### Vulcan Carbon XC72R Pretreatment

Vulcan XC72R (Cabot, USA) was chosen as a standard support material for electrocatalysts. A pretreatment step with H_2_O_2_ was conducted to improve its hydrophilicity and wettability by carbon surface oxidation.^47^ Typically, 1 g of Vulcan XC72R was dispersed in 100 mL of 30% H_2_O_2_ solution (30% H_2_O_2_, p.a., ISO, Carl Roth, Germany) and continuously stirred at 500 rpm for ∼6 h at ∼70 °C. Afterward, the suspension was diluted, filtered in a Büchner funnel, washed with ultrapure H_2_O (18.2 MΩcm, Merck Millipore, USA), and dried in a furnace (Heraeus, Germany) for 24 h at 60 °C.

### Electrochemical Erosion of PtCu/C

For a typical synthesis of PtCu/C, ∼75 mL of 1 M KOH (85%, Grüssing, Germany) solution was prepared, to which different amounts of CuSO_4_ (CuSO_4_·5 H_2_O, ≥99.5%, p.a., ACS, ISO, Carl Roth, Germany) were added to reach a CuSO_4_ concentration of 0.4 mM–0.9 mM in 1 M KOH. After ultrasonication of the solution for ∼1 h for better dispersion, ∼30 mg of H_2_O_2_-pretreated Vulcan XC72R was added to the solution, and the suspension was subsequently placed in an ultrasonication bath (SONOREX SUPER RK 31, Bandelin, Germany) under continuous stirring for ∼1 h to achieve homogeneous dispersion of the carbon support. Two Pt wires (Ø 0.25 mm, 99.99%, MaTecK, Germany) were immersed in the suspension at a distance of ∼4 cm under continuous stirring and ultrasonication. An alternating potential signal (VSP-300, BioLogic, France) with a frequency of 200 Hz and an amplitude of 10 V–25 V between the two wires was applied via large amplitude sinusoidal voltammetry (LASV). The erosion process started instantly and led to the formation of PtCu NPs attached to the carbon support. In contrast to cyclic voltammetry, the double-layer capacitive current is not subject to a sharp transition at the reverse potentials due to the signal’s sinusoidal waveform. Aiming for a Pt loading of ∼20 wt % requires the erosion of approximately 7.5 mg of Pt wires, corresponding to a length of ∼3.5 mm per wire (typically corresponding to ∼1 h). The exact Pt amount was specified by comparing the mass of the Pt wires before and after erosion through an analytical balance, simultaneously determining the end of the synthesis procedure. After the desired Pt weight fraction was obtained, the suspension was left stirring for ∼12 h at room temperature. The continuous stirring should promote sufficient mixing and, therefore, attachment of PtCu nanoparticles on the carbon support. In addition, it is supposed to minimize uncontrolled particle mobility and catalyst degradation during final electrochemical testing by minimizing their surface free energy during extended stirring directly after completion of the synthesis. Filtering, washing with ultrapure H_2_O, and drying in the furnace in air for ∼12 h at 60 °C yielded the PtCu/C electrocatalyst. In addition, a Pt/C catalyst was synthesized as a reference according to the above-described methodology via electrochemical erosion to illustrate the impact of the alloying with Cu to derive a more active ORR electrocatalyst. The synthesis was conducted in 1 M KOH without the addition of CuSO_4_ using an alternating potential signal frequency of 200 Hz and an amplitude of 15 V.

### TEM

The morphology, structure, and chemical composition of the PtCu nanoparticles on carbon support were studied on the nanoscale with a Cs image-corrected transmission electron microscope TITAN Themis 60–300 Cubed (Thermo Fisher Scientific, USA), operated at an acceleration voltage of 300 kV. Scanning TEM-energy dispersive X-ray spectroscopy (STEM-EDX) elemental mapping was conducted at 300 kV (large view fields up to ∼240 nm) and 60 kV to mitigate electron beam damage while analyzing small view fields (∼35 nm). STEM-EDX spectroscopy was performed with a Super-X detector, and elemental maps and chemical composition were acquired and processed in the Velox software utilizing a standard Cliff–Lorimer (*K*-factor) quantification method. EDX maps were created in the form of atomic % of elements of interest. Samples for TEM analysis were prepared from a dispersed solution made of PtCu/C powder and pure methanol, which was drop-casted onto a copper TEM grid coated with a carbon holey membrane. The FFT analysis was conducted using the CrysTBox tool.^68^

### Powder-XRD

XRD provided the crystallographic and structural properties of the synthesized Pt/C and PtCu/C-nanostructured catalysts. The samples were illuminated with a Cu–K_α_ (λ = 1.5406 Å) source with an integrated Ni-based filter by a Rigaku MiniFlex 600 C (Rigaku Corporation, Japan). The device recorded diffraction patterns in a slow-scanning mode with a 1° min^–1^ step velocity from 5° to 135°. The data analysis was conducted by applying the full-profile Rietveld method as implemented in the program FullProf.^69^ Vulcan carbon background-subtracted diffraction patterns were modeled assuming isotropic peak broadening. The instrumental resolution was determined from Si reference measurements.

### XPS

XPS spectra of the PtCu/C catalyst with the highest ORR activity were collected on a SPECS setup (SPECS XR50 X-ray source, SPECS PHOIBOS 150 hemispherical analyzer, and SPECS spectrometer; Specs, Germany) using a monochromatized Al–K_α_ source (1486.7 eV). Thirty μL of the catalyst ink was drop-casted on a glassy carbon disk and dried. XPS spectra of the thin catalyst ink film on glassy carbon were recorded before and after electrochemical characterization. All spectra were acquired in an ultrahigh vacuum chamber at an operating pressure below 1 × 10^–9^ mbar. The data was analyzed with the Casa XPS software (Version 2.3.24PR1.0). The binding energy scale correction was done using the C=C peak of the C 1s spectrum at 284.0 eV.

### ICP-MS

ICP-MS of the PtCu/C catalysts was conducted on a PerkinElmer NexIon 350D (PerkinElmer, USA) ICP-MS instrument to obtain an accurate quantification of the Pt and Cu elemental amount. ^195^Pt and ^64^Cu were used as target masses for the analytes and ^103^Rh for the internal standard. Analyte quantification was carried out in standard mode with a correction equation to account for polyatomic interferences. External calibration was performed in the range of 0 μg L^–1^–100 μg L^–1^. Each sample was measured with five measurement replicates, a dwell time of 50 ms, and an integration time of 750 ms. For the sample preparation, ∼1 mg was weighed with a micro scale, digested with freshly prepared aqua regia, heated to 80 °C, diluted with ultrapure H_2_O, filtered over silica, and again diluted to meet the calibrated concentration range. Each mass (Pt and Cu) was measured five times and averaged to yield the final ICP-MS result. The relative standard deviation for Pt was determined between 0.8% and 2.0%, while that of Cu was determined between 1.8% and 3.0%.

### Catalyst Ink Preparation

The typical ink ratio consisted of ∼10 mg of finely ground catalyst powder, 3.6 mL of ultrapure H_2_O, 1.446 μL of isopropanol (puriss. p.a., ACS reagent, ≥99.8%, Sigma-Aldrich, USA), and 30 μL of Nafion dispersion (5 wt % in lower aliphatic alcohols and water, Sigma-Aldrich, USA). Ultrasonication for 5 min ensured a homogeneous mixing of the ink components.

### Electrochemical Evaluation

All PtCu/C catalysts were characterized electrochemically in a three-electrode configuration, consisting of the catalyst coating, a curled Pt wire, and a mercury/mercurous sulfate electrode (MMS, SI Analytics, Germany) as the working (WE), counter (CE), and reference electrode (RE), respectively. All glassware was cleaned prior to the measurements via a 3:1 mixture of H_2_SO_4_ (96%, Suprapur, Merck, Germany) and H_2_O_2_ to remove organic residues, with subsequent washing by boiled ultrapure H_2_O to remove any sulfate residues. An OrigaTrod electrode rotator (OrigaLys ElectroChem SAS, France) and a suitable electrode tip functioned as the RDE. For the RRDE experiments, a Pine MSR electrode rotator (Pine Instruments, USA) was used. The RDE electrode tip comprised a glassy carbon disk (Ø 5 mm) encased in polyether ether ketone (PEEK), providing a geometrical surface area of 0.196 cm^2^. The RRDE tip contained a Pt ring and a glassy carbon disk (Ø 5 mm) encased in polytetrafluoroethylene (PTFE), providing a geometrical surface area of 0.196 cm^2^. If not mentioned otherwise, the recorded current densities, displayed in the CVs and ORR polarization curves, were normalized to the geometrical surface area. Prior to each measurement, the tips were polished with 1.0 μm, 0.3 μm, and 0.05 μm grain-sized alumina paste (MicroPolishTM, Buehler, USA). Typically, an aliquot of 10–15 μL of the catalyst ink was drop-casted on the freshly polished glassy carbon tip to ensure perfectly smooth catalyst layers covering the entire glassy carbon RDE area. This corresponded to Pt mass loadings of approximately 2.4 to 5.0 μg_Pt_ cm^–2^. The hot air of a heat gun (D5950 Remington, Germany) dried the drop-casted aliquot under constant stirring to ensure homogeneous coatings. For electrochemical characterizations, CV techniques were applied in either Ar-saturated (5.0, Westfalen, Germany) or O_2_-saturated (4.5, Westfalen, Germany) 0.1 M HClO_4_ solutions (70%, extra pure, Acros, Germany). First, the catalyst was cycled with a scan rate of 50 mV s^–1^ between 0.06 and 1.2 V vs RHE in Ar-saturated 0.1 M HClO_4_ after purging with Ar for 30 min until a stable CV was obtained to remove potential surface contaminations and to electrochemically activate the catalyst. The last cycle was used to determine the ECSA via H_UPD_. The integrated charge of the hydrogen adsorption and desorption peaks between ∼0.1 V and ∼0.4 V vs RHE was normalized to the assumed total charge of 210 μC cm^–2^ for a monolayer of hydrogen adsorbed on Pt. Normalizing the ECSA to the Pt weight loading on the glassy carbon electrode results in the synthesized catalysts’ SSA. For determining the ORR activity, the 0.1 M HClO_4_ solution was purged with O_2_ for 30 min to ensure saturation conditions. Thereafter, CVs were recorded with a scan rate of 10 mV s^–1^ and a rotation rate of 1600 rpm in a potential window from 0.2 to 1.1 V vs RHE. The potentials were *iR*-corrected by determining the uncompensated resistance via electrochemical impedance spectroscopy (EIS) by a frequency perturbation in the range of 200 kHz to 10 Hz with a perturbation amplitude of 25 mV at various potentials between 0.8 and 0.9 V vs RHE. The EIS measurements were conducted in O_2_-saturated 0.1 M HClO_4_ with a rotation rate of 1600 rpm. In addition, a recorded CV in Ar atmosphere at a scan rate of 10 mV s^–1^ was subtracted from the ORR polarization curve to correct for (pseudo)­capacitive current contributions. The ORR activities were evaluated at 0.9 V vs RHE by deriving the kinetic current from the polarization curve according to the equation: |*i*_k_| = (|*i*| × |*i*_L_|)/(|*i*_L_| – |*i*|), where *i*_k_, *i*_L_, and *i* represent the kinetic current, diffusion-limited current, and measured current, respectively. MAs and SAs were determined by normalizing the kinetic current at 0.9 V vs RHE to the Pt mass on the glassy carbon and the ECSA, respectively. All potentials are given with respect to the RHE scale if not stated otherwise. The conversion from MMS to RHE potential scale was done by calibrating the MMS reference electrode in H_2_-saturated (5.0, Westfalen, Germany) 0.1 M HClO_4_ by measuring the intercept of the hydrogen oxidation/evolution curve with the *x*-axis. The intercept refers to the conversion potential specific to the MMS reference electrode and 0.1 M HClO_4_ electrolyte solution used in our experiments (typically ∼ −0.72 V vs MMS). The same procedure was followed for the reference Pt/C catalyst, synthesized in 1 M KOH without CuSO_4_ with a potential amplitude of 15 V. For each catalyst sample, at least two individual measurements were conducted following the protocol described above. The sample produced with a 0.54 mM CuSO_4_ concentration and a 15 V potential amplitude was characterized by six independent measurements to ensure an accurate depiction of its electrochemistry. The corresponding standard deviations of the SSA, SA, and MA are given in the discussion and the Supporting Information.

## Supplementary Material



## Notes

Comparative ICP-MS results of synthesized PtCu/C catalysts (Figure S1); exemplary AST CVs (Figure S2). HR-TEM images, corresponding FFT patterns, and lattice spacings before and after AST (Figure S3) with discussion; TEM images with corresponding SAED and FFT patterns before and after AST (Figure S4); HR-TEM images with FFT patterns of different locations on two different PtCu NPs (Figures S5 and S6) with corresponding *d*-spacings (Tables S1 and S3); *d*-spacings of Pt and Cu from the literature (Table S2); STEM-EDX elemental maps of PtCu/C before AST (Figure S7); fitted XPS of C 1s and O 1s core level regions (Figure S8); fitted XPS of Cu 2p core level regions (Figure S9); table with all identified XPS peaks and locations (Table S4); XRD patterns of all synthesized PtCu/C samples (Figure S10) with discussion; fitted and background-subtracted XRD patterns (Figure S11); graphical illustration of lattice parameters, lattice contraction, and average crystallite sizes (Figure S12); overview of XRD results (Table S5); CVs of all synthesized PtCu/C samples in Ar-sat. 0.1 M HClO_4_ with corresponding SSAs (Figure S13); overview of Pt and Cu mass loadings by ICP-MS (Table S6); overview of electrocatalytic properties (Table S7); discussion of RRDE and Koutecký–Levich analysis; graphical illustration of Koutecký–Levich analysis (Figure S14); overview of numerous PtCu catalysts from the literature (Table S8); Bar graph for comparison of MAs (Figure S15); ORR polarization curves of all synthesized PtCu/C samples (Figure S16); derived MAs and SAs of all synthesized PtCu/C samples (Figure S17) with discussion; bar graphs for comparison of the stability of PtCu/C with commercial Pt/C_TKK_ catalyst (Figure S18) with discussion (PDF)
