# Supplementary material for: Synthesis of PtCu/C Nanostructured Electrocatalysts for the Oxygen Reduction Reaction via One-Step Electrochemical Erosion
Source: ACS Appl Mater Interfaces. 2026 Jan 6;18(2):4210–24. doi: 10.1021/acsami.5c22270 (PMC12828716; doi:10.1021/acsami.5c22270)
Supplement: Supplementary file 1 [file am5c22270_si_001.pdf]

Supporting Information

Synthesis of PtCu/C Nanostructured Electrocatalysts for the Oxygen Reduction Reaction *via* One-Step Electrochemical Erosion

Peter M. Schneider[1], Eva Kolíbalová[2], Jhonatan Rodriguez-Pereira[2,3], Theophilus K. Sarpey[1,4], Christian M. Schott[1], Elena L. Gubanova[1], Pavan Kumar Chennam[2], Anatoliy Senyshyn[5], Christine Benning[6], Martin Elsner[6], Jan M. Macak[2,3,*], Aliaksandr S. Bandarenka[1,7,*]

1 - Physics of Energy Conversion and Storage, Technical University of Munich, James-Franck-Str. 1, 85748 Garching, Germany

2 - Central European Institute of Technology, Brno University of Technology, Purkynova 123, 61200 Brno, Czech Republic

3 - Center of Materials and Nanotechnologies, University of Pardubice, Nam. Cs. Legii 565, 53002 Pardubice, Czech Republic,

4 - Materials Research Department, GSI Helmholtzzentrum für Schwerionenforschung GmbH, Planckstraße 1, 64291 Darmstadt, Germany

5 - Heinz Maier-Leibnitz Zentrum (MLZ), Technische Universität München, Lichtenbergstr. 1, 85748 Garching, Germany

6 - Chair of Analytical Chemistry and Water Chemistry, School of Natural Sciences, Technical University of Munich, 1Lichtenbergstr. 4, 85748 Garching, Germany

7 - Catalysis Research Center TUM, Ernst-Otto-Fischer-Str. 1, 85748 Garching, Germany

* Corresponding Authors: E-mails: bandarenka@ph.tum.de (A.S. Bandarenka),

Jan.Macak@ceitec.vutbr.cz (J.M. Macak)

KEYWORDS: Oxygen reduction reaction, Nanoparticles, Electrochemical erosion, Platinum alloys, Electrocatalysis

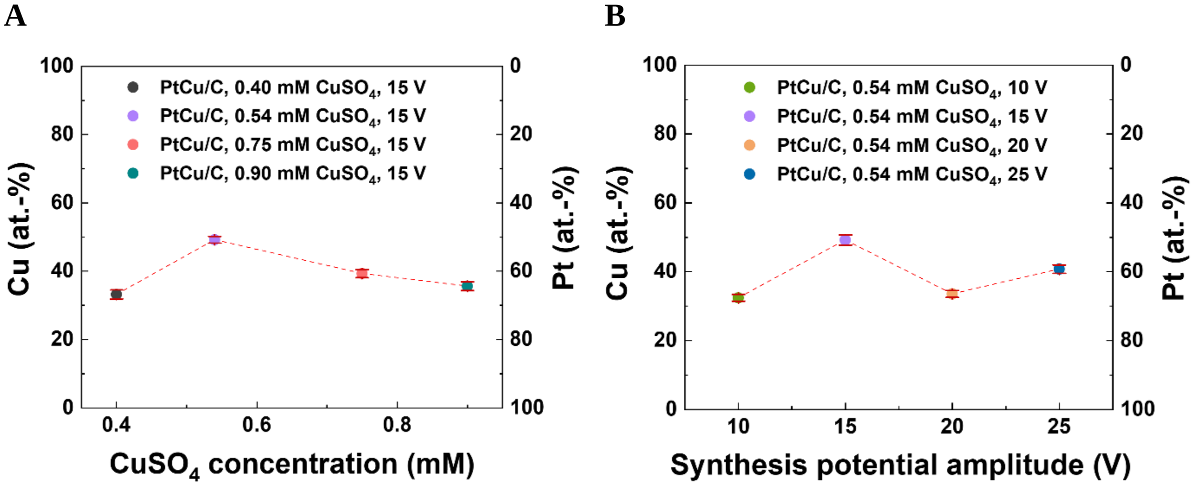

Figure S1. Atomic ratio between Pt and Cu of the PtCu/C electrocatalysts synthesized by electrochemical erosion depending on (A) the $CuSO_4$ concentration (0.40 mM, 0.54 mM, 0.75 mM, and 0.90 mM) and (B) the applied synthesis potential amplitude (10 V, 15 V, 20 V, and 25 V). All results were acquired via inductively coupled plasma mass spectrometry (ICP-MS). For the sample with 0.54 mM $CuSO_4$ concentration and 15 V potential amplitude, the same ICP-MS data point was used in both displays. The dashed line serves as a guide for the eye. The standard deviation errors are indicated in red.

Electrochemical activation and stability testing. Since electrocatalyst materials are exposed to harsh conditions during their operation in, e.g., fuel cells, it is important to gain information about their stability and performance after electrocatalytic cycling in acidic media. Information about eventual changes in NP size, composition, structure, and electrocatalytic performance is crucial for their potential applicability. Therefore, preliminary accelerated stress tests (ASTs) were performed for all synthesized PtCu/C catalysts, which have been employed in electrocatalytic studies of novel catalyst nanostructures before [1,2]. After initial cycling of the RDE catalyst coating in Ar-saturated 0.1 M $HClO_4$ until a stable cyclic voltammogram (CV) was achieved, the ORR activity was determined in O_2-saturated 0.1 M $HClO_4$ at 1600 rpm with a scan rate of 10 mV s^{-1}. Afterward, accelerated stress tests (ASTs) were conducted by cycling the catalysts in O_2-saturated 0.1 M $HClO_4$ at 1600 rpm for 1000 cycles in a potential window ranging from 0.6 V to 1.0 V vs. the reversible hydrogen electrode (RHE) with a scan rate of 100 mV s^{-1} to get preliminary information about the stability of the PtCu/C catalysts. An exemplary AST CV is shown in **Figure S2**.

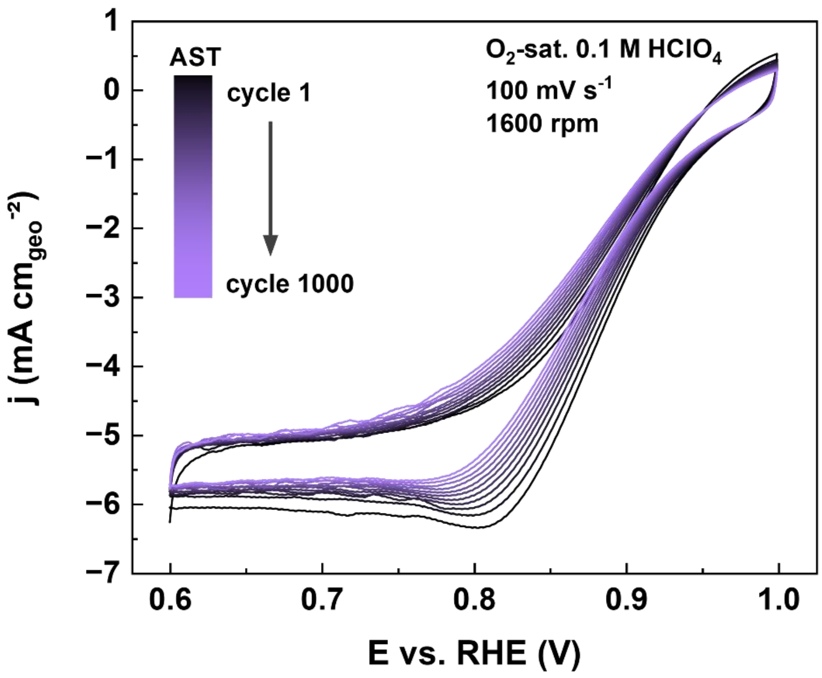

Figure S2. Exemplary accelerated stress test CV in O_2-saturated 0.1 M $HClO_4$ at a rotation speed of 1600 rpm and 100 mV s^{-1} scan rate for PtCu/C synthesized in 1 M KOH and 0.54 mM $CuSO_4$ with a potential amplitude of 15 V. 1000 cycles have been performed in the range of 0.6 V to 1.0 V vs. RHE. The figure shows the first cycle and every 100th cycle thereafter. The recorded current was normalized to the geometric surface area of the glassy carbon electrode (0.196 cm^2).

Figures S3C-E and **S3F-H** show individual nanoparticles before and after electrochemical cycling (AST), respectively. The Fast Fourier transformation (FFT) patterns match the crystallographic structure response of the [011] zonal axis of an *fcc* lattice structure. In general, the identified lattice spacings, shown in **Figures S3E** and **H**, match those of the pure Pt *fcc* lattice from the database (PDF card number 04-0802). Nevertheless, they deviate slightly from the theoretical values for $(1\bar{1}1)$, (200), and $(0\bar{2}2)$, corresponding to ~ 225.9 pm, ~ 195.6 pm, and ~ 138.3 pm, respectively. Before the AST, $(1\bar{1}1)$ and (200) were evaluated to be ~ 241 pm and ~ 197 pm, respectively, while after AST, $(1\bar{1}1)$, (200), and $(0\bar{2}2)$ corresponded to ~ 227 pm, ~ 201 pm, and ~ 132 pm, respectively. These deviations from the theoretical values could stem from lattice strain either through surface distortions by the electrochemical erosion approach itself [7], or through alloying and subsequent acid leaching-induced dealloying during the conducted AST, which introduces lattice strain through the rearrangement of surface Pt alloys. Precisely this alteration of the lattice spacings between before and after AST could indicate a

structural change of the catalyst surface over cycling through acid leaching-induced dealloying of the PtCu nanoparticles [3], as the increasing deviation of the $(1\bar{1}\bar{1})$ and $(0\bar{2}\bar{2})$ planes would imply a higher degree of strain. Furthermore, the identification of an *fcc* structure with slight deviations from the theoretical pure Pt lattice spacings in the FFT patterns indicates alloy formation through the incorporation of Cu into the Pt crystal structure as a mixed solid solution. The formation of ordered intermetallic structures (e.g., PtCu, Pt_3Cu, Cu_3Pt) would lead to additional superlattice responses, so-called forbidden reflections. Additional selected area electron diffraction (SAED) and FFT measurements before and after electrochemical cycling, shown in **Figures S4A-C** and **D-E**, respectively, exhibit numerous reflection signals. However, no presented intermetallic Pt-Cu crystal phase (e.g., PtCu, $PtCu_3$, Pt_7Cu) matches all detected reflections adequately, based on the TEM imaging results. It is worth noting that the finite PtCu nanostructures in this study significantly limit the resolution and structural response visible in FFT images, complicating the analysis. Therefore, we hypothesize the formation of a disordered mixed solid-solution alloy of Pt and Cu, with a distorted *fcc* Pt crystal lattice, rather than the formation of an ordered Pt-Cu phase. This interpretation is most plausible given the relatively harsh synthesis conditions during electrochemical erosion. Moreover, the structural analysis *via* TEM did not reveal significant changes between before and after AST except for an alteration of the lattice spacings, which could stem from acid-leaching-induced dealloying, as described above. As a result, the synthesized PtCu NPs can be assumed to exhibit good structural stability upon electrochemical cycling.

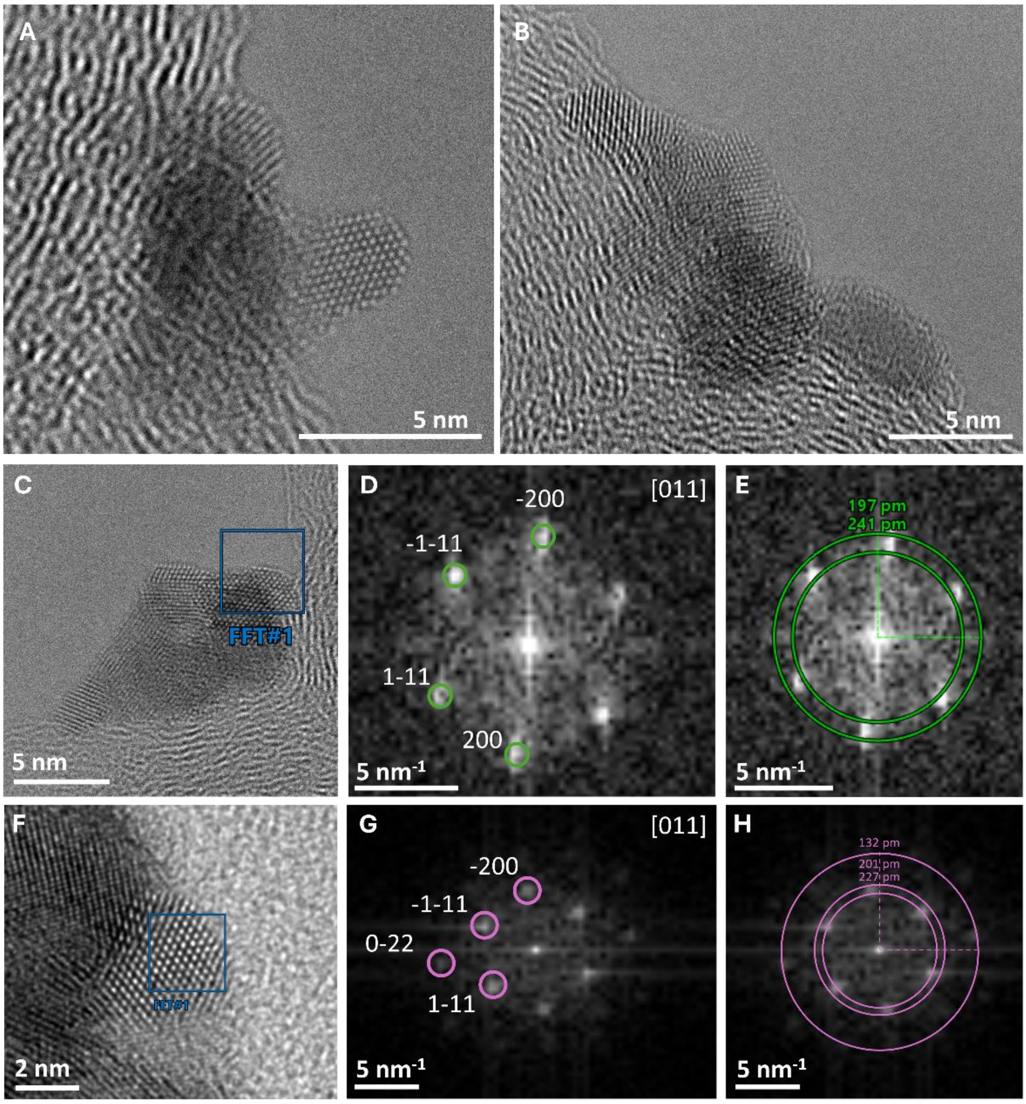

Figure S3. (A) and (B) HR-TEM images of selected nanoparticles supported on carbon before electrochemical cycling. (C) HR-TEM image of an individual nanoparticle before electrochemical cycling with (D) corresponding FFT pattern and (E) lattice spacings. (F) HR-TEM image of an individual nanoparticle after AST with (G) corresponding FFT pattern and (H) lattice spacings. The detected lattice planes in (D) and (G) were assigned to the Pt *fcc* lattice.

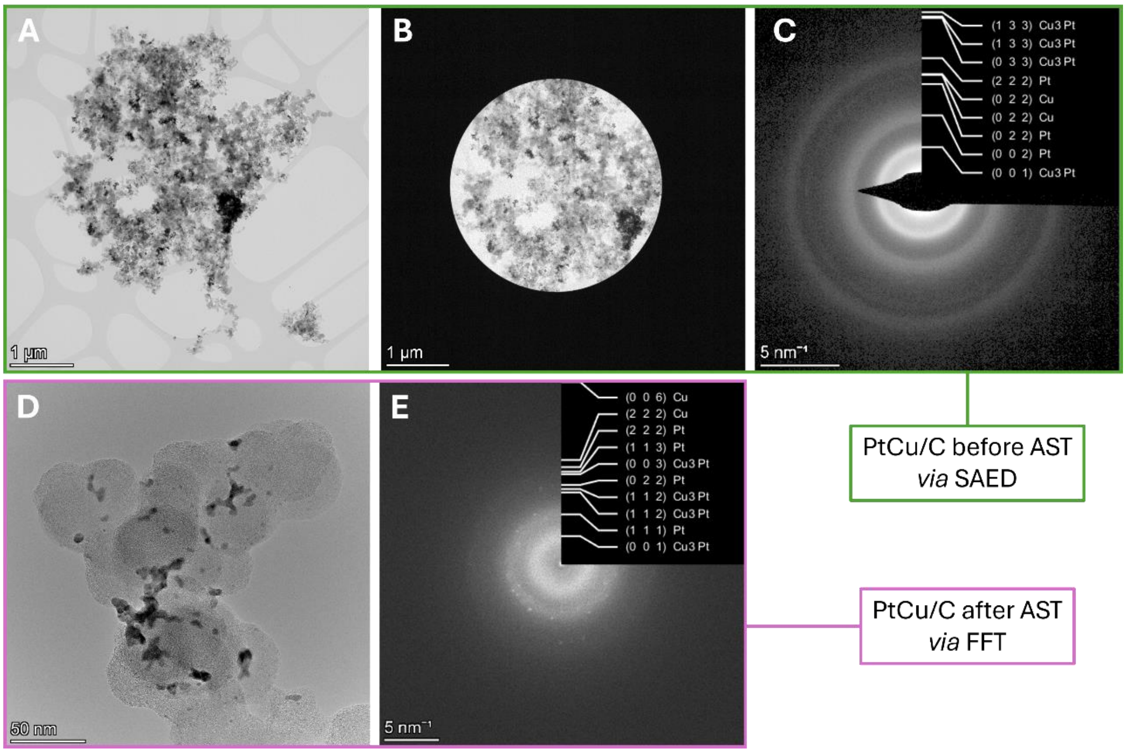

Figure S4. (A) Overview image of the PtCu/C before electrochemical characterization. (B) TEM image showing an encircled volume of a SAED aperture for diffraction analysis. (C) The resulting electron diffraction pattern with rings corresponding to possible lattice planes of Pt-Cu crystal phases. (D) and (E) TEM image and corresponding FFT pattern with possible Pt-Cu crystal structures of PtCu nanoparticles after AST, respectively.

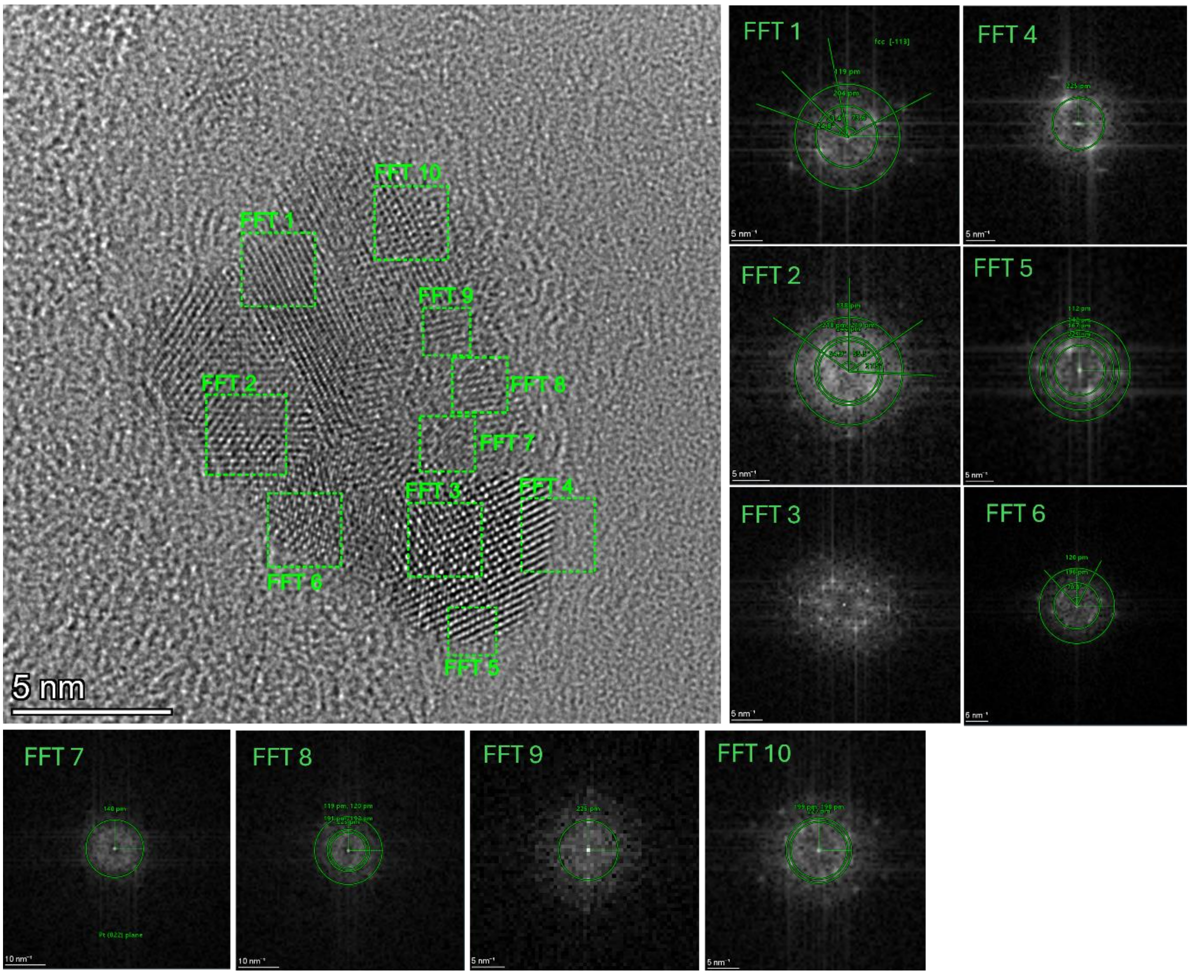

Figure S5. HR-TEM image of the nanoparticle from Figure 2A with the marked areas for FFT analysis and the corresponding FFT reflection patterns and determined d-spacings.

Table S1. Identified lattice spacings and possible lattice orientations based on the FFT analysis. The number of the FFTs equals the numbers shown in Figure S5. The corresponding nanoparticle area in Figure 2A is included as well.

FFT #	Area in Figure 2A	Lattice spacing [pm]	Possible lattice orientation
1	2a	204	Pt (220)
		119	
		91	
2	1	244	Pt (11$\bar{1}$)
		219	
		138 - 144	
3	5b	-	
4	5a	225	Pt (111)
5	5a	225	Pt (111)
		167	
		142	
6	4	196	-
		120	
7	3a	140	Pt (220)
8	3b	225	Pt (111)
		191 - 192	
		119 - 120	
9	3c	225	Pt (111)
10	2b	227	-
		198 - 199	

Table S2. Lattice planes and corresponding d-spacings in pm for Pt and Cu, obtained from the database.

Pt (101103.cif)		Cu (410568.cif)	
Lattice plane	d-spacing [pm]	Lattice plane	d-spacing [pm]
(111)	227.7	(111)	208.7
(200)	197.2	(200)	180.7
(220)	139.4	(220)	127.9
(113)	118.9	(113)	109.0
(222)	113.9	(222)	104.3
(400)	98.6	(400)	90.3
(133)	90.5	(133)	82.9
(024)	88.2	(024)	80.8
(224)	80.5		

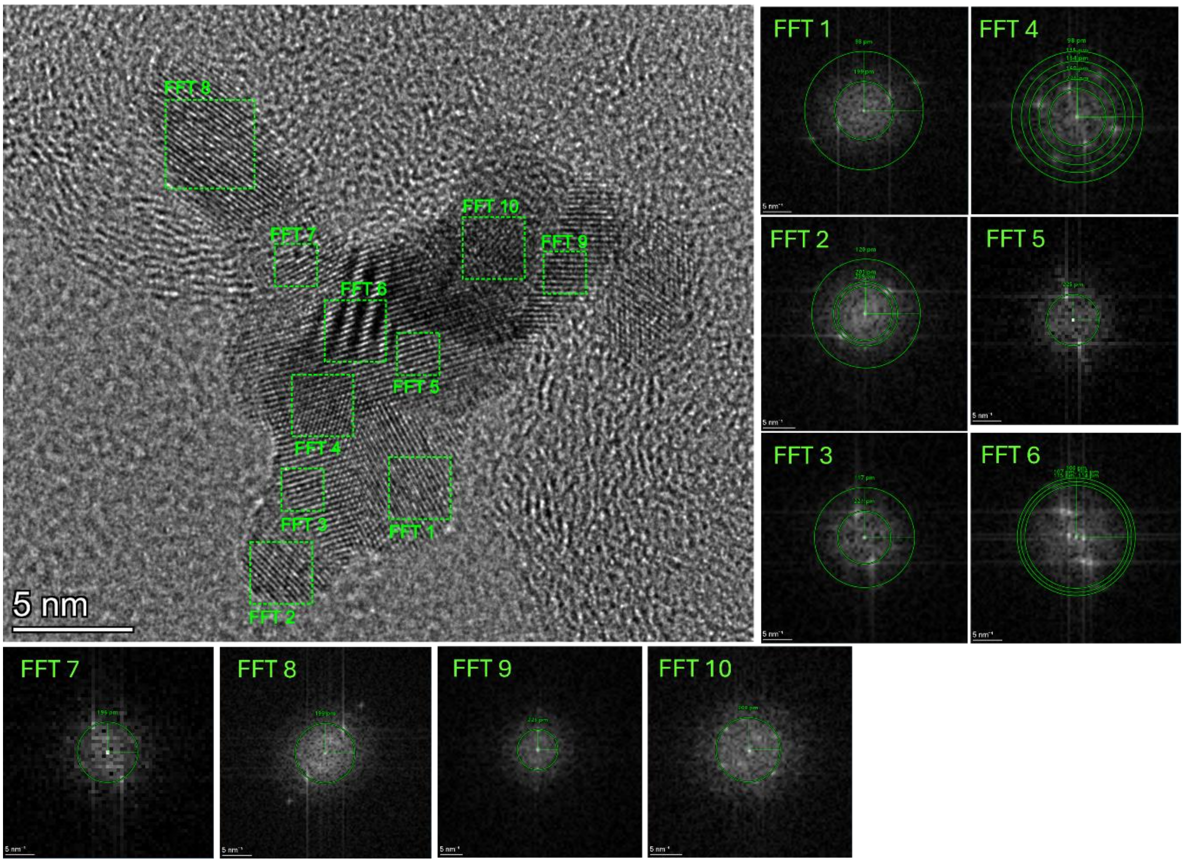

Figure S6. HR-TEM image of the nanoparticle from Figure 2D with the marked areas for FFT analysis and the corresponding FFT reflection patterns and determined d-spacings.

Table S3. Identified lattice spacings and estimated lattice orientations based on the FFT analysis. The number of the FFTs equals the numbers shown in Figure S6.

FFT #	Lattice spacing [pm]	Possible lattice orientation
1	98	Pt (200) or Cu (111)
	199	
2	120	Pt (200) or Cu (111)
	203	
	239	
3	117	Pt (111)
	221	
4	97	CuPt trigonal phase
	115	
	135	
	167	
	228	
5	225	Pt (111)
6	100	Moire pattern
	106 - 107	
	114 - 115	
7	196	Pt (200) or Cu (111)
8	199	Pt (200) or Cu (111)
9	223	Pt (111)
10	200	Pt (200) or Cu (111)
	198 - 199	

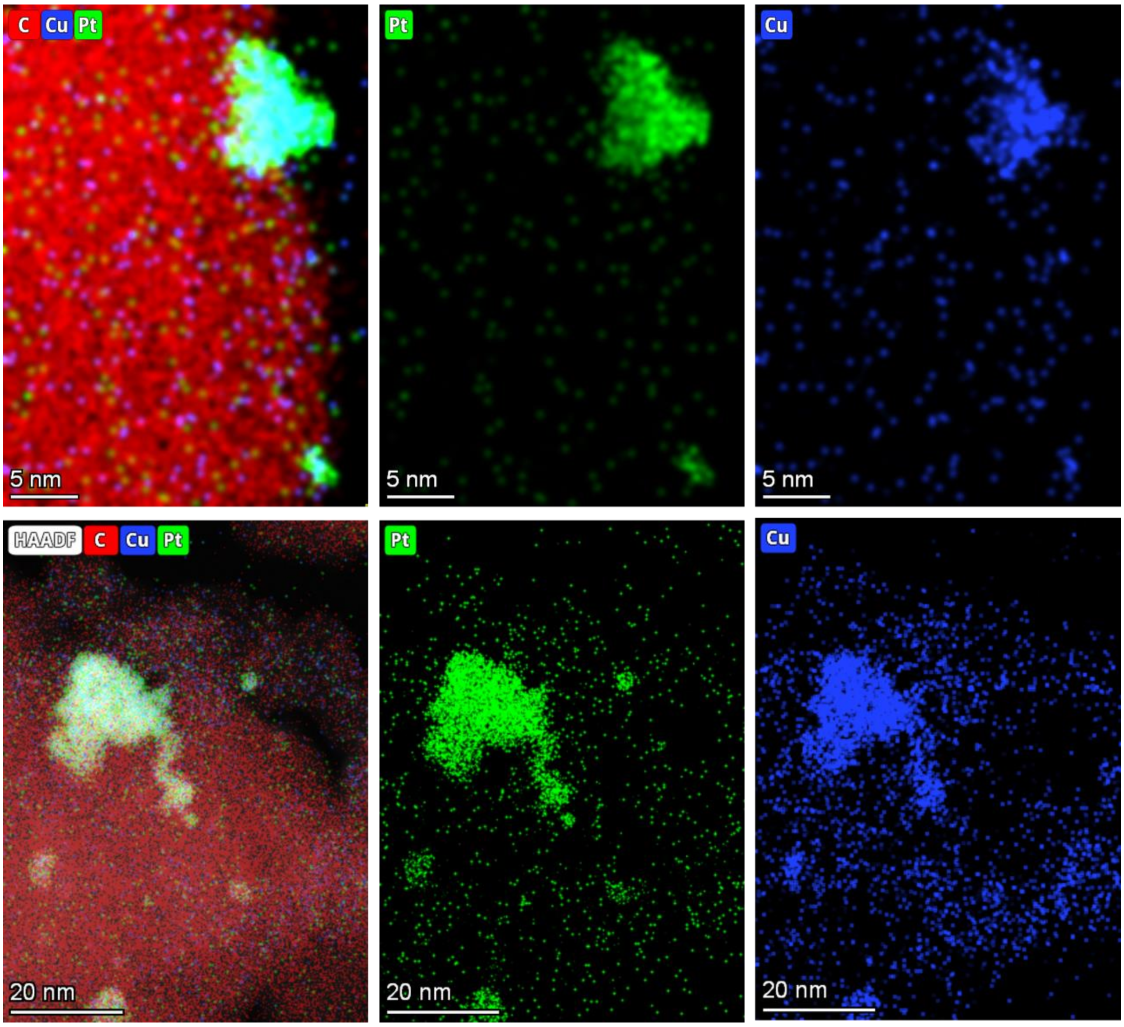

Figure S7. STEM-EDX elemental maps of the PtCu/C catalyst synthesized in 1 M KOH and 0.54 mM CuSO4 with 15 V potential amplitude before electrochemical cycling (AST). Colors show the fluorescence bands of Pt-L, Cu-L, and the combined bands of Pt-L, Cu-L, and C-K for two individual areas.

XPS analysis of C 1s and O 1s. The fitted C 1s and O 1s spectra are displayed in **Figure S6** before (top) and after (bottom) electrochemical cycling. The C 1s regions show typical peaks of porous Vulcan® carbon (C-(C,H), C=C). The oxygen-related peaks may arise from the carbon support pretreatment with H_2O_2, as described in the experimental section. Furthermore, fluorine-related peaks (C-F, C=F) were identified, which can be ascribed to the use of Nafion in the catalyst ink for the coatings used in XPS measurements. The electrochemical cycling had only a minor impact on the XPS signals, i.e., a reduced (C-C,H) peak. The O 1s spectra showed minor changes over cycling. Generally, the oxygen signal is mainly ascribed to carbon and Nafion. Nevertheless, parts of the oxygen signal could stem from the presence of metal hydroxides (Pt-OH and Cu-OH), overlapping with the C=O component.

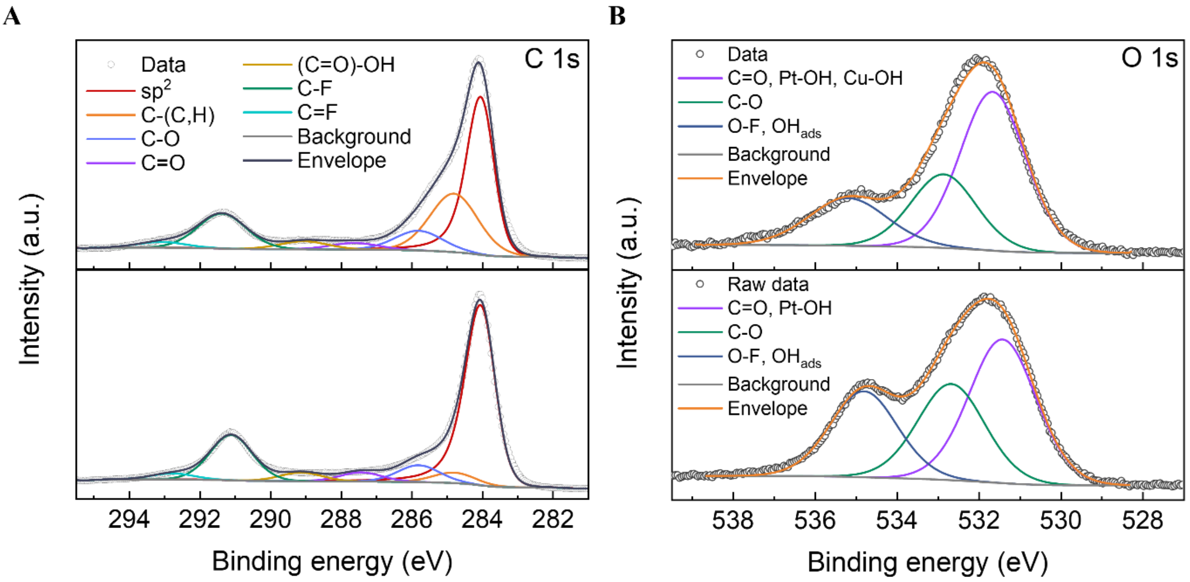

Figure S8. Fitted XPS spectra of the (A) C 1s and (B) O 1s core level regions of the PtCu/C catalyst sample synthesized in 1 M KOH and 0.54 mM $CuSO_4$ with a 15 V potential amplitude before (top) and after (bottom) electrochemical cycling.

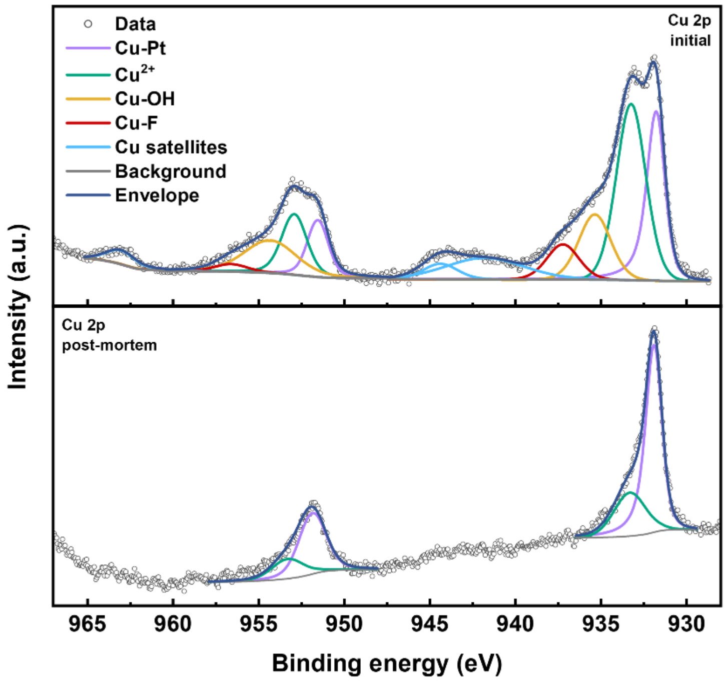

Figure S9. Fitted XPS spectra of the Cu 2p core-level regions of the PtCu/C catalyst sample synthesized in 1 M KOH and 0.54 mM $CuSO_4$ with a 15 V potential amplitude before (top) and after (bottom) electrochemical cycling, depicting the complete XPS binding energy range.

Table S4. Identified XPS signals and their peak positions according to the performed fitting for the Pt 4f, Cu 2p, C 1s, and O 1s core-level regions before and after electrochemical cycling of the PtCu/C catalyst synthesized in 1 M KOH and 0.54 mM $CuSO_4$ with a 15 V potential amplitude.

Before electrochemical cycling		After electrochemical cycling	
Identified peak	Peak position [eV]	Identified peak	Peak position [eV]
C=C (sp^2)	284.0	C=C (sp^2)	284.0
C-(C,H)	284.8	C-(C,H)	284.8
C-O	285.8	C-O	285.8
C=O	287.6	C=O	287.4
(C=O)-OH	289.1	(C=O)-OH	289.1
C-F	291.4	C-F	291.1
C=F	293.1	C=F	292.8
C=O, Pt/Cu-OH	531.6	C=O, Pt-OH	531.3
C-O	532.9	C-O	532.6
O-F, OH_{ads}	535.2	O-F, OH_{ads}	534.8
Pt-Cu (7/2)	70.8	Pt-Cu (7/2)	70.8
Pt-Cu (5/2)	74.1	Pt-Cu (5/2)	74.1
Pt^0 (7/2)	71.3	Pt^0 (7/2)	71.3
Pt^0 (5/2)	74.6	Pt^0 (5/2)	74.6
Pt^{2+} (7/2)	72.7	Pt^{2+} (7/2)	72.5
Pt^{2+} (5/2)	76.1	Pt^{2+} (5/2)	75.9
Pt^{4+} (7/2)	74.8	Pt^{4+} (7/2)	74.6
Pt^{4+} (5/2)	78.2	Pt^{4+} (5/2)	77.9
Pt-Cu	931.7	Cu-Pt	931.8
Cu^{2+}	933.2	Cu^{2+}	933.1
Cu-OH	935.3	-	-
Cu-F	937.2	-	-

XRD analysis. The lattice parameters were estimated using the Rietveld refinement method, assuming the *fcc* lattice structure of Pt. The lattice contraction/strain was estimated from the lattice parameters of the synthesized PtCu NPs and the Pt reference [4]. The calculated lattice parameters and lattice contractions are illustrated in **Figure S12**. Interestingly, the lattice parameter decreases almost linearly with increasing $CuSO_4$ concentration during the synthesis, as evident from **Figure S10A**, indicating an increasing amount of Cu being incorporated into the lattice structure of the PtCu nanoparticles. The lattice parameter/lattice contraction values for $CuSO_4$ concentrations of 0.75 mM and 0.90 mM are almost identical, which could indicate an emerging saturation effect. In contrast, altering the potential amplitude during the synthesis

did not reveal a significant change in the lattice parameters, and, therefore, the lattice contraction. Besides, all samples feature a smaller lattice parameter than the reference Pt/C catalyst and Pt from the database, demonstrating the successful application of electrochemical erosion as a method for fabricating PtCu alloy nanoparticles. **Table S5** summarizes all values and parameters deduced from Rietveld refinement of the X-ray diffraction data. However, these findings contradict the relationship between the Cu content and the $CuSO_4$ concentration during synthesis, as determined by ICP-MS (shown in **Figure S1**), which revealed a maximum Cu content for the sample synthesized with a 0.54 mM $CuSO_4$ concentration. Accordingly, this sample should exhibit the smallest lattice parameter and the largest lattice contraction among all samples, assuming a full incorporation of the Cu. The reason for the apparent discrepancy between the ICP-MS and XRD results could originate from potential residuals of the electrochemical erosion process (e.g., CuO, $Cu(OH)_x$, $CuSO_4$, etc.), which are still detected as "Cu" by ICP-MS, but are not taken into account in the XRD analysis by Rietveld refinement modeling. Small XRD reflections visible at ~ 35.69° in **Figure S10** provide evidence in support of this hypothesis. For three PtCu/C samples (with potential amplitudes and $CuSO_4$ concentrations: 15 V + 0.54 mM $CuSO_4$, 15 V + 0.75 mM $CuSO_4$, and 25 V + 0.54 mM $CuSO_4$), noticeable CuO peaks could be observed. Interestingly, these three samples exhibit the highest Cu contents according to the ICP-MS measurements. The increasing $CuSO_4$ concentration during the synthesis thus led to an increased amount of Cu incorporation into the PtCu nanoparticles, as evident from the XRD results. However, the ICP-MS results could occasionally be influenced by residual compounds originating from the electrochemical erosion process. It is worth noting that both ICP-MS and XRD serve as bulk characterization techniques, yielding a global average that may not necessarily reflect the compositions of individual nanoparticles. This is evident from the STEM-EDX analysis of individual nanostructures displayed in **Figures 3** and **S7**, which indeed reveal a ~ 1:1 Pt:Cu atomic ratio. In the following discussion of the electrocatalytic activities, the XRD results will serve as the primary reference for the catalysts' average composition, as Rietveld refinement offers a more precise description of the PtCu nanoparticle structure than ICP-MS. The latter nevertheless provides valuable information regarding the Pt loading on the Vulcan® carbon support, essential for quantifying the electrocatalytic performance.

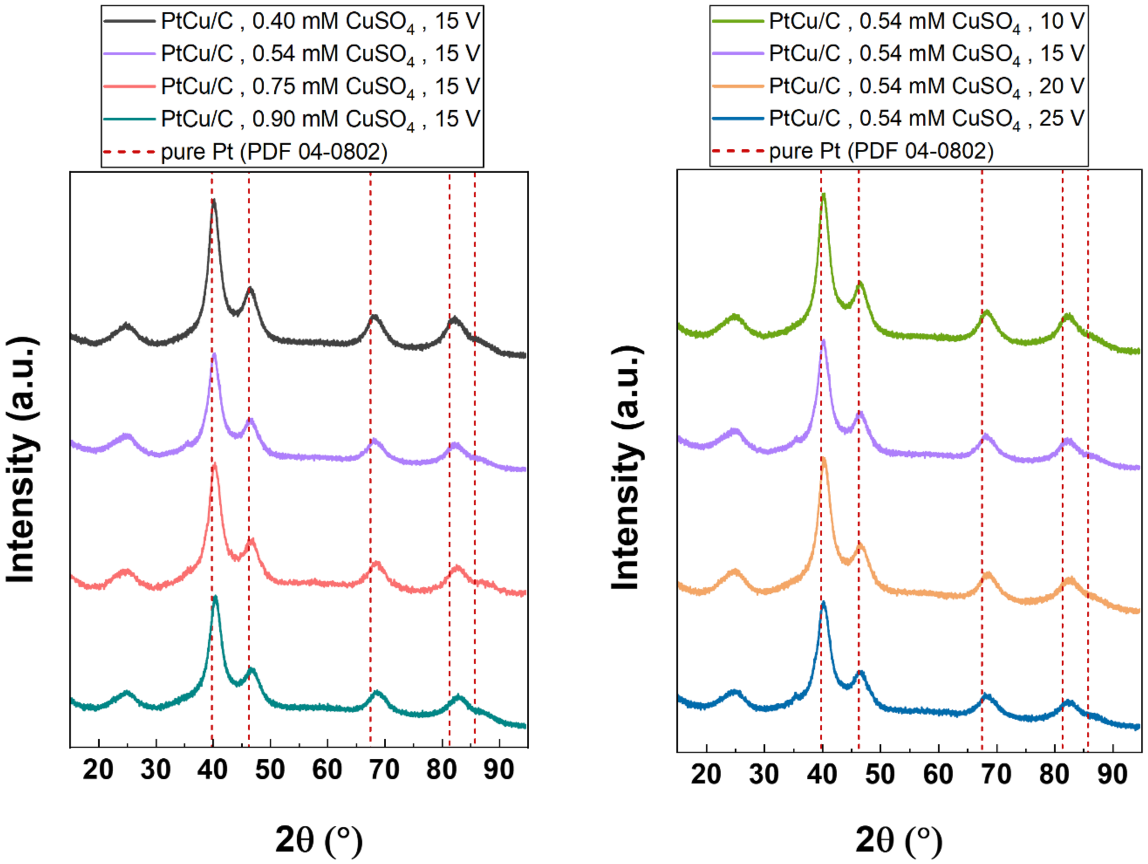

Figure S10. XRD patterns of the synthesized PtCu/C catalysts in dependence of (A) the $CuSO_4$ concentration (0.4 mM – 0.9 mM $CuSO_4$) and (B) the potential amplitude (10 V – 25 V). The peak positions of pure Pt, adapted from PDF card number 04-0802, are indicated by red-dotted vertical bars. The same data of the sample with 0.54 mM $CuSO_4$ concentration and 15 V potential amplitude was used for both studies.

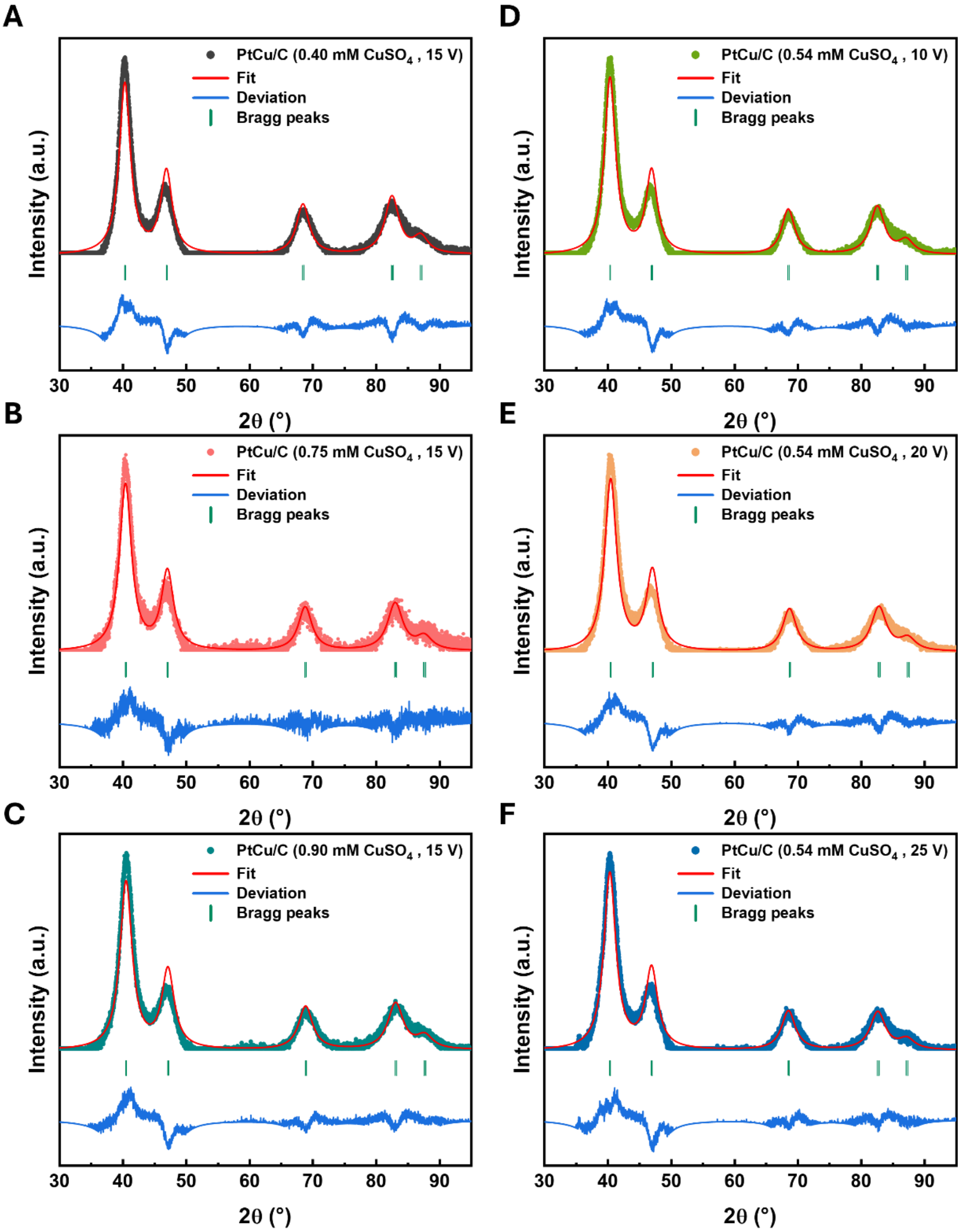

Figure S11. Background-subtracted XRD pattern of the PtCu/C samples synthesized with (A), (B), (C) different $CuSO_4$ concentrations at 15 V or with (D), (E), and (F) different potential amplitudes using 0.54 mM $CuSO_4$. The red curves show the refined theoretical profile according to the Rietveld method. The blue profiles represent the deviation between the measured and modeled XRD profiles. The green tickmarks illustrate the identified Bragg peaks.

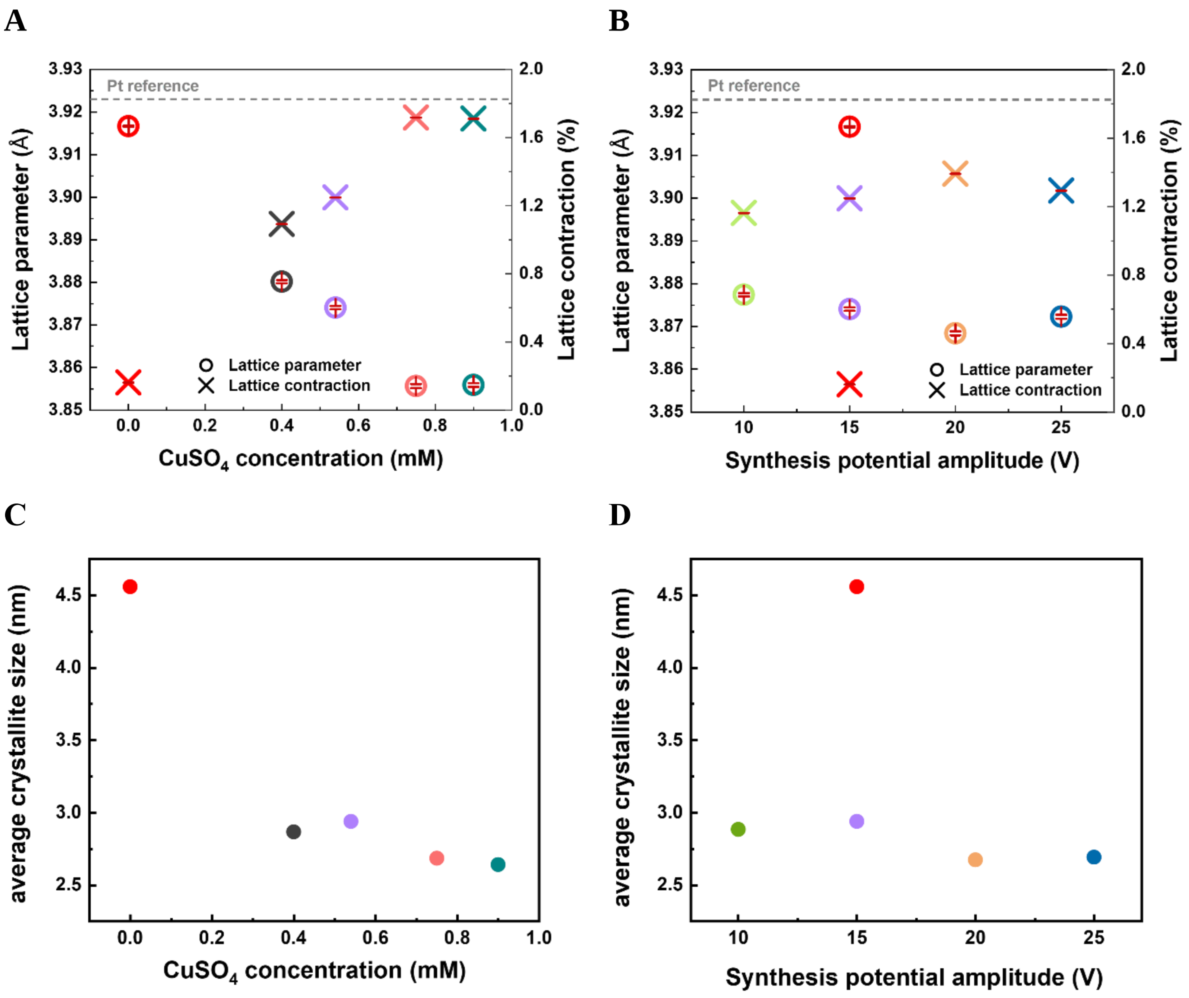

Figure S12. Estimated lattice parameters and lattice contractions for the synthesized PtCu/C catalysts in dependence on (A) the $CuSO_4$ concentration (0.4 mM – 0.9 mM $CuSO_4$) and (B) the potential amplitude (10 V – 25 V). The horizontal dashed lines represent the estimated lattice parameter of pure Pt from PDF card number 04-0802. Average apparent crystallite size in dependence on (C) the $CuSO_4$ concentration (0.4 mM – 0.9 mM $CuSO_4$) and (D) the potential amplitude (10 V – 25 V). The red circle represents the reference Pt/C catalyst synthesized without $CuSO_4$. The data point of the sample with a concentration of 0.54 mM $CuSO_4$ and a potential amplitude of 15 V was used for both representations. Fitting errors are included as red vertical error bars. The degree of anisotropy for the average apparent crystallite sizes was determined as ~ 0.001 nm for all samples.

Table S5. Overview of chemical and structural parameters of the synthesized PtCu/C electrocatalysts. Atomic percentages of Cu from ICP-MS. Estimated lattice parameters, contractions relative to pure Pt (PDF card number 04-0802), and average crystallite sizes as measured by XRD. The data point of the sample with a concentration of 0.54 mM $CuSO_4$ and a potential amplitude of 15 V was added twice (as sample "0.54 mM" and "15 V") for better comparison. Errors from the Rietveld refinement fitting are included for the lattice parameter. The errors of the lattice contraction are negligible (< 0.1 %). The degree of anisotropy for the average apparent crystallite sizes was determined as ~ 0.001 nm for all samples.

sample	Lattice parameter [Å]	Contraction vs pure Pt [%]	av. crystallite size [nm]
Pt	3.923 ± 0.001	0	-
Pt/C	3.917 ± 0.001	0.16	4.6
0.40 mM	3.880 ± 0.001	1.09	2.9
0.54 mM	3.874 ± 0.001	1.25	2.9
0.75 mM	3.856 ± 0.001	1.72	2.9
0.90 mM	3.856 ± 0.001	1.71	2.6
10 V	3.877 ± 0.001	1.16	2.9
15 V	3.874 ± 0.001	1.25	2.9
20 V	3.868 ± 0.001	1.39	2.7
25 V	3.872 ± 0.001	1.29	2.7

SSA trends: Comparing the PtCu/C samples produced with different potential amplitudes, a decrease in SSA was observed with increasing potential amplitude. This follows the expected trend of smaller SSA for larger Pt NPs produced at higher potential amplitudes *via* the electrochemical erosion approach, as experimentally demonstrated by Garlyyev *et* al. [2]. Therefore, the decreasing trend of the SSA for the PtCu/C catalysts could be explained by an increasing nanostructure size with increasing potential amplitude. While TEM characterization of all synthesized catalyst systems was not applicable in this study, Rietveld refinement results in **Figure S12D** and **Table S5** reveal no trend in the average apparent crystallite size. However, these crystallite sizes could be correlated with the crystalline domain sizes within individual nanoparticles according to **Figure 2.** Consequently, different potential amplitudes could still lead to small differences in the overall nanoparticle dimensions, and therefore, the SA, despite unchanged average crystalline sizes, assuming the identified grains are mostly unaffected by the variation in the synthesis potential amplitude.

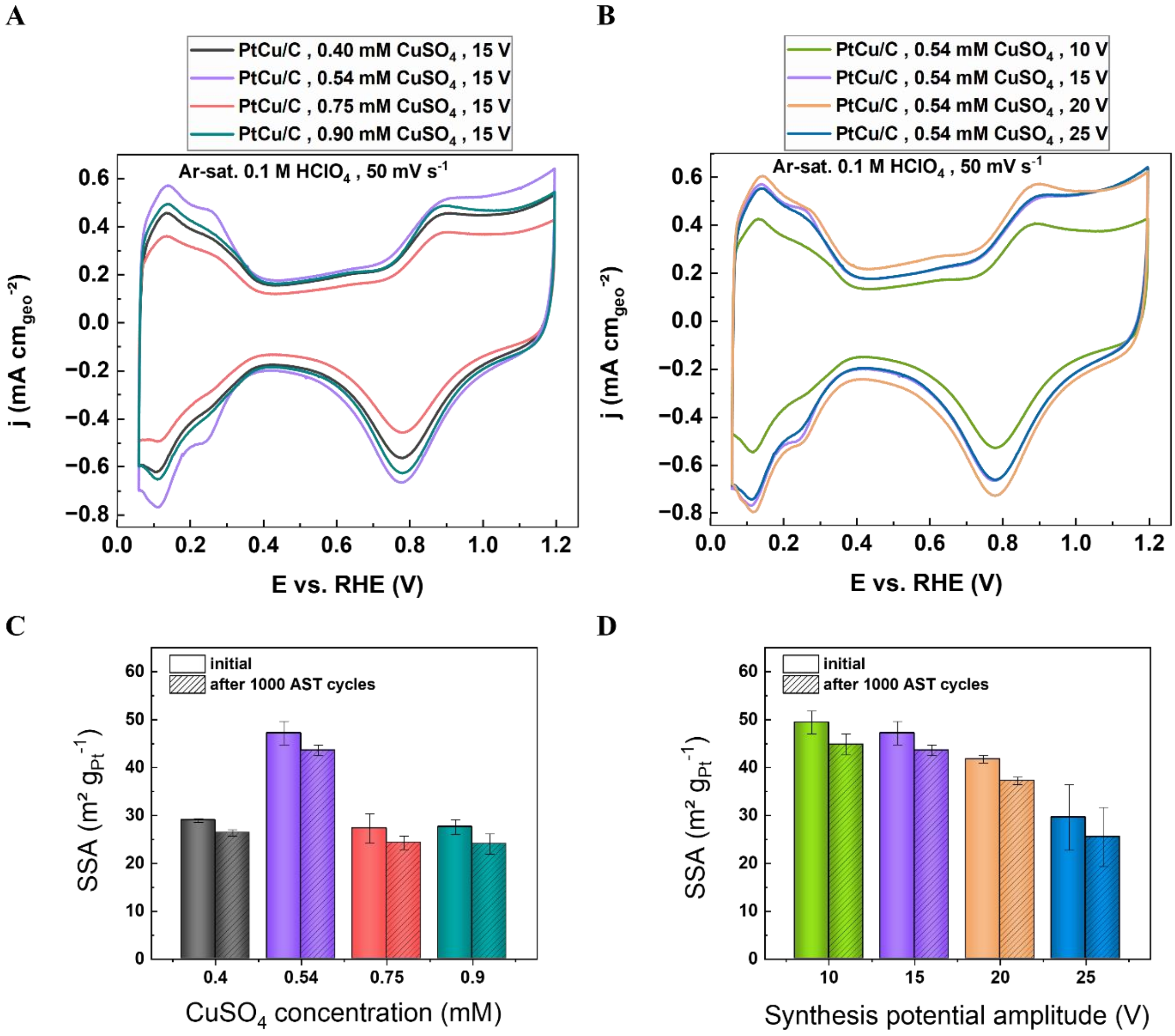

Figure S13. Typical CVs of the synthesized PtCu/C electrocatalysts with (A) varying $CuSO_4$ concentration (0.40 mM – 0.90 mM) and (B) varying potential amplitude (10 V – 25 V) in Ar-saturated 0.1 M $HClO_4$ at 50 mV s^{-1} scan rate. All currents were normalized to the geometrical area of the glassy carbon electrode (0.196 cm^2). (C) and (D) display of the calculated SSAs by dividing the ECSAs determined from the HUPD peaks of (A) and (B), respectively, by the corresponding Pt mass loadings from **Table S3**. The data point of the sample with a concentration of 0.54 mM $CuSO_4$ and a potential amplitude of 15 V was used for both representations.

Table S6. Overview of the mass loadings of Pt and Cu for all synthesized PtCu/C catalysts via ICP-MS. It is noted that the applied potential amplitude was fixed to 15 V for the series of experiments addressing the influence of the $CuSO_4$ concentration, while the concentration was fixed to 0.54 mM for the series of experiments exploring the potential amplitude effect. The sample with a concentration of 0.54 mM $CuSO_4$ and a potential amplitude of 15 V was used for both studies.

$CuSO_4$ concentration study			Synthesis potential amplitude study		
Sample	Pt mass loading [wt.-%]	Cu mass loading [wt.-%]	Sample	Pt mass loading [wt.-%]	Cu mass loading [wt.-%]
0.40 mM	[illegible]	[illegible]	10 V	[illegible]	[illegible]
0.54 mM	[illegible]	[illegible]	15 V	[illegible]	[illegible]
0.75 mM	[illegible]	[illegible]	20 V	[illegible]	[illegible]
0.90 mM	[illegible]	[illegible]	25 V	[illegible]	[illegible]

Table S7. Overview of the evaluated electrocatalytic properties of the synthesized PtCu/C catalysts. The SA and MA values were calculated by normalizing the kinetic current at 0.9 V vs. RHE by the ESCA and Pt weight loading, respectively. For each measurement, at least two individual electrochemical measurements were performed. Six individual electrochemical measurements were performed for PtCu/C synthesized in 1 M KOH and 0.54 mM $CuSO_4$ concentration with a 15 V potential amplitude.

sample	Pt loading [wt.-%]	SSA [$m^2\ g_{Pt}^{-1}$]	SA [$mA\ cm^{-2}$]	MA [$A\ mg_{Pt}^{-1}$]
15 V, 0.40 mM	19	29.0 ± 0.4	2.54 ± 0.13	0.66 ± 0.03
15 V, 0.54 mM	12	47.2 ± 2.5	2.48 ± 0.16	1.16 ± 0.07
15 V, 0.75 mM	21	27.3 ± 3.1	2.51 ± 0.26	0.69 ± 0.10
15 V, 0.90 mM	22	27.6 ± 1.6	2.37 ± 0.03	0.65 ± 0.07
10 V, 0.54 mM	17	49.4 ± 2.4	2.35 ± 0.14	1.16 ± 0.13
20 V, 0.54 mM	19	41.8 ± 0.8	2.78 ± 0.32	1.16 ± 0.11
25 V, 0.54 mM	18	29.6 ± 6.8	3.00 ± 0.62	0.89 ± 0.31

RRDE experiments. For this type of measurement, the same procedure was followed as for the RDE measurements described in the experimental part. We made use of the concept of the RRDE setup, where potential H_2O_2 produced during the ORR is oxidized at the Pt ring. The disk potential was swept between 0.2 V and 1.1 V vs. RHE while the Pt ring was held constant at 1.2 V vs. RHE. The H_2O Faradaic efficiency can be calculated as $\left(1-\frac{I_R}{n I_D}\right)100\%$, where I_D, I_R, and n denote the disk current, ring current, and collection efficiency, respectively [5]. The collection efficiency was determined by recording CVs of the ferrocyanide/ferricyanide half reactions at

different rotation speeds. Dividing the obtained ring current by the disk current provides the collection efficiency n of the RRDE setup, which was ~ 22% in this study. The number of transferred electrons n can be calculated via $n = 4\frac{i_D}{i_D + \frac{i_R}{N}}$, which is shown as the dashed gray curve in **Figure S14C**.

Koutecký-Levich analysis. To gain information about the reaction kinetics of our PtCu/C catalyst, a series of experiments in O_2-saturated 0.1 M $HClO_4$ was performed. Polarization curves at a scan rate of 10 mV s^{-1} were recorded at different rotation speeds. The reciprocal value of the ORR current was plotted against the reciprocal square root of the rotation speed at different electrode potentials from the ORR onset region to the mass diffusion limitation region. This representation yields a straight line, which can be described by the Koutecký-Levich equation: $\frac{1}{i} = \frac{1}{i_K} + \frac{1}{B\omega^{1/2}}$, where i is the recorded current, i_K is the kinetic current, equal to the intercept with the y-axis, and $1/B$ is the slope of the line. Here, the proportionality coefficient $B = 0.62 n F A D_{O_2}^{2/3} \nu^{-1/6} C$, where n is the number of transferred electrons, F is the Faraday constant, A is the geometrical surface, D_{O_2} is the diffusion coefficient of molecular oxygen, ν is the kinematic viscosity of the electrolyte, and C is the concentration of the oxygen in the bulk electrolyte [6]. As shown in **Figure S14C**, n, derived from the Koutecký-Levich analysis, is close to the expected value (n = 4) but slightly deviates depending on the potential range. According to the literature, this method has only limited application for the ORR despite its widespread use because the ORR is not a one-step reaction and is not irreversible, which results in n being slightly dependent on the rotation speed ω.[6] Nevertheless, it proves the transfer of ~ 4 electrons and an efficient reduction reaction of O_2 to H_2O.

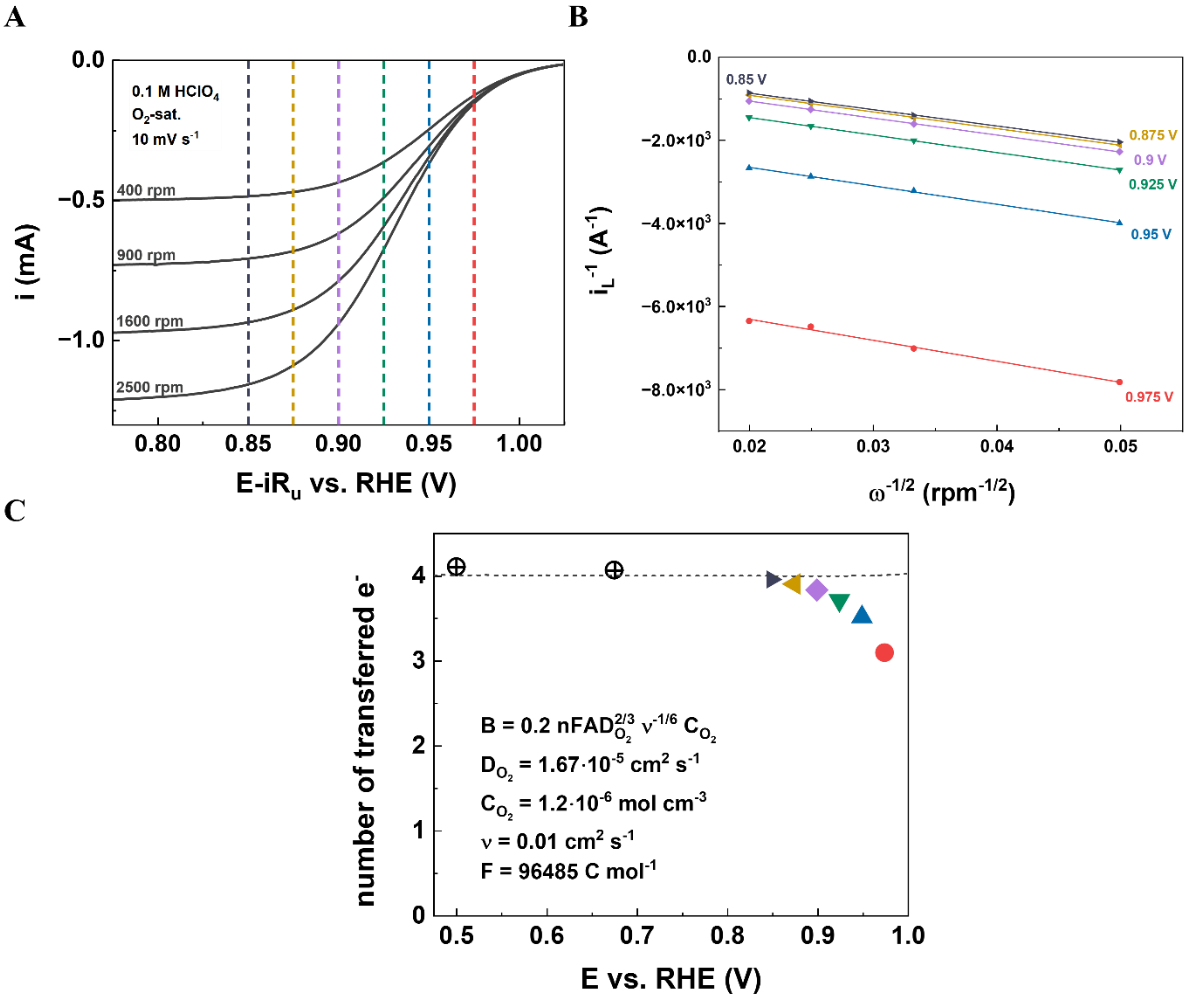

Figure S14. (A) Typical polarization curves of the PtCu/C, synthesized in 1 M KOH and 0.54 mM $CuSO_4$ with a potential amplitude of 15 V, at different rotation speeds in O_2-saturated 0.1 M $HClO_4$ at 10 mV s^{-1} scan rate. The vertical dashed lines represent the potentials at which the Koutecký-Levich analysis was performed. (B) Koutecký-Levich lines by plotting the reciprocal current values from (A) over the inverse square root values of the rotation rate for selected potentials. (C) Number of transferred electrons calculated from (B) plotted against the RHE scale. The colors of (C) and (B) correspond to the colors indicated in (A). The dashed grey curve shows the calculated electron transfer number via the RRDE method. The cross-marked circles in (C), corresponding to the analysis at 0.7 and 0.5 V vs. RHE, are not shown in (B) for better visualization.

Table S8. Overview table of the electrocatalytic properties of various PtCu catalyst samples from the literature. Sample names were taken from the original publications. SSAs, SAs, and MAs are given together with the literature reference numbers. We included commercial Pt/C (Tanaka and E-TEK) and previous works (Pt/C_{TD} and Pt_xPr/C) via electrochemical erosion for comparison. * denotes one of the best-performing samples fabricated within this work (0.54 mM $CuSO_4$ concentration + 15 V potential amplitude). Standard deviations are included where available.

Catalyst	SSA [m^2 g_{Pt}^{-1}]	SA [mA cm^{-2}]	MA [A mg_{Pt}^{-1}]	Ref.
Pt/C_{Tanaka}	68 ± 2	0.61 ± 0.03	0.42 ± 0.02	[7]
Pt/C_{E-TEK}	71	0.24	0.17	[8]
$PtCo/C_{Umicore}$	62	0.39	0.24	[9]
$PtCo/C_{Tanaka}$	49	1.14	0.56	[10]
Pt/C_{TD}	46 ± 8	1.62 ± 0.32	0.71 ± 0.02	[7] previous work
Pt_xPr/C	36 ± 2	1.95 ± 0.14	0.70 ± 0.04	[1] previous work
PtCu/C*	47 ± 3	2.48 ± 0.16	1.16 ± 0.07	* this work
$Pt_{75}Cu_{25}$	55	0.42 ± 0.09	0.23 ± 0.02	[3]
$Pt_{50}Cu_{50}$	59	0.76 ± 0.18	0.45 ± 0.14	[3]
$Pt_{25}Cu_{75}$	74	0.76 ± 0.10	0.56 ± 0.01	[3]
Cu_3Pt/C	54	0.85	0.46	[11]
$PtCu_3$	58 ± 6	0.78	0.45 ± 0.04	[12]
PtCu	45 ± 6	0.84	0.38 ± 0.06	[12]
Pt_3Cu	40 ± 2	0.58	0.23 ± 0.02	[12]
$PtCu_2$	33	1.29	0.42	[13]
PtCuN/KB	64	1.18	1.15	[14]
PtCu/KB	58	1.40	0.81	[14]
PtCu-800	117 ± 6	1.85 ± 0.22	2.16 ± 0.16	[15]
Cu_3Pt/C	58	1.1	0.64	[16]
PtCu/C	20 ± 2	3.4 ± 0.3	0.68 ± 0.07	[17]
$PtCu_3$	45	2.5	1.1	[18]
$PtCu_{0.9}$	41 ± 4	0.55 ± 0.05	0.22	[19]
$Pt_{20}Cu_{20}Co_{60}$	111	0.44	0.49	[20]
$Pt_{0.25}Cu_{0.75}$	73	0.76 ± 0.01	0.55 ± 0.01	[21]
Pt_2Cu/C	53	1.44	0.75	[22]
$Pt_2CuW_{0.25}/C$	33	1.03	0.34	[22]
$PtCu_{0.24}$	43	2.03 ± 0.13	0.88 ± 0.05	[23]
Pt_2Cu/C	-	-	0.51	[24]
$PtCu_{0.3}$	47	2.31	1.09	[25]
$Pt_{0.25}Cu_{0.75}$	31	1.48	0.46	[26]
$Pt_{0.5}Cu_{0.5}$	53	1.52	0.80	[26]
$Pt_{0.75}Cu_{0.75}$	43	1.39	0.59	[26]
PtCu-250	47 ± 3	2.30 ± 0.13	1.06 ± 0.10	[27]
$Pt_{55}Cu_{45}$	63	1.29	0.82	[28]
$Pt_{92}Cu_8$	58	0.58	0.34	[28]
$Pt_{76}Cu_{24}$	37	1.27	0.47	[29]
$Pt_{52}Cu_{48}$	31	0.99	0.31	[29]

$Pt_{28}Cu_{72}$	29	0.88	0.26	[29]
PtCu	28	4.25	1.20	[30]
PtCu	55	5.98	3.26	[31]
O-PtCuNF/C	53	4.69 ± 0.21	2.47 ± 0.10	[32]
D-PtCuNF/C	56	2.12 ± 0.05	1.18 ± 0.05	[32]
PtCu	66	1.24	0.82	[33]

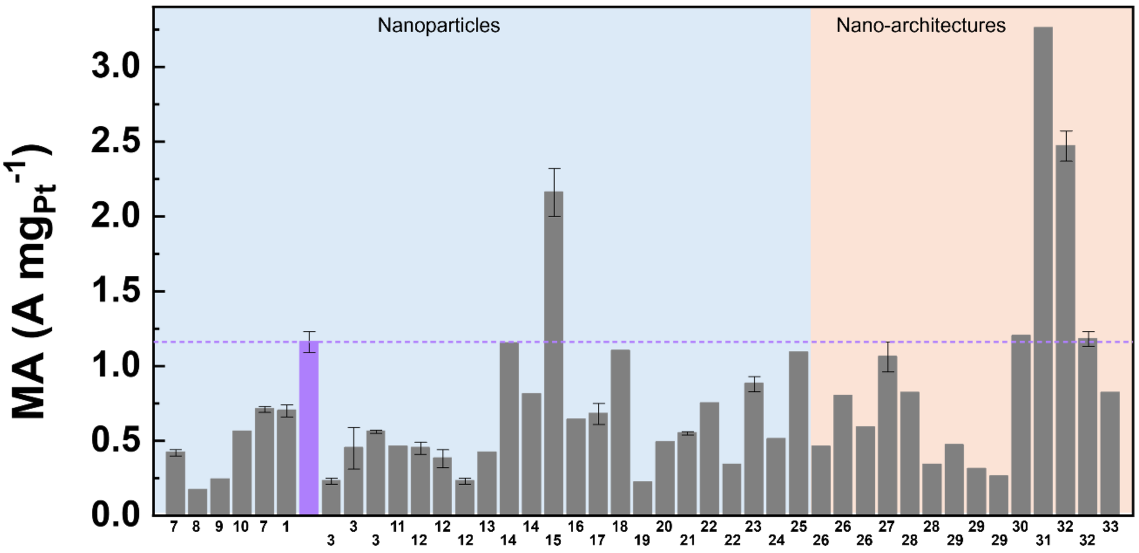

Figure S15. Bar graph comparing the MA of PtCu/C catalyst from this work (0.54 mM $CuSO_4$ concentration + 15 V potential amplitude, denoted as *) with previously reported PtCu electrocatalysts. The numbers underneath the bars represent the literature references as listed in **Table S5**. We included commercial Pt/C (Tanaka and E-TEK) and previous works (Pt/C_{TD} and Pt_xPr/C) via electrochemical erosion for comparison. Standard deviations were added upon availability in the original publications.

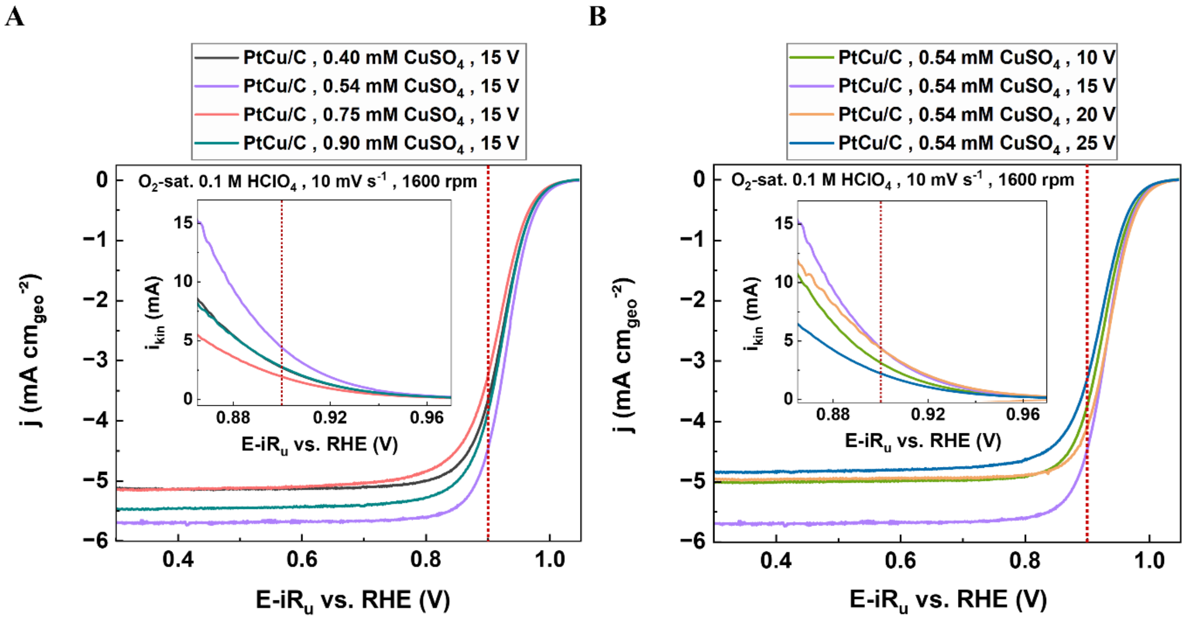

Figure S16. Typical iR-corrected anodic polarization curves of the PtCu/C catalysts synthesized with (A) varying $CuSO_4$ concentration (0.40 mM – 0.90 mM) and (B) varying potential amplitude (10 V – 25 V) in O_2-saturated 0.1 M $HClO_4$ at 10 mV s^{-1} scan rate and 1600 rpm rotation speed. The polarization curves were corrected from (pseudo)capacitive current contributions by subtracting a CV recorded in Ar-saturated conditions with a 10 mV s^{-1} scan rate. The inset shows the calculated kinetic currents. The vertical red bars mark the potential at which the ORR activities were determined (0.9 V vs. RHE). All currents were normalized to the geometrical area of the glassy carbon electrode (0.196 cm^2).

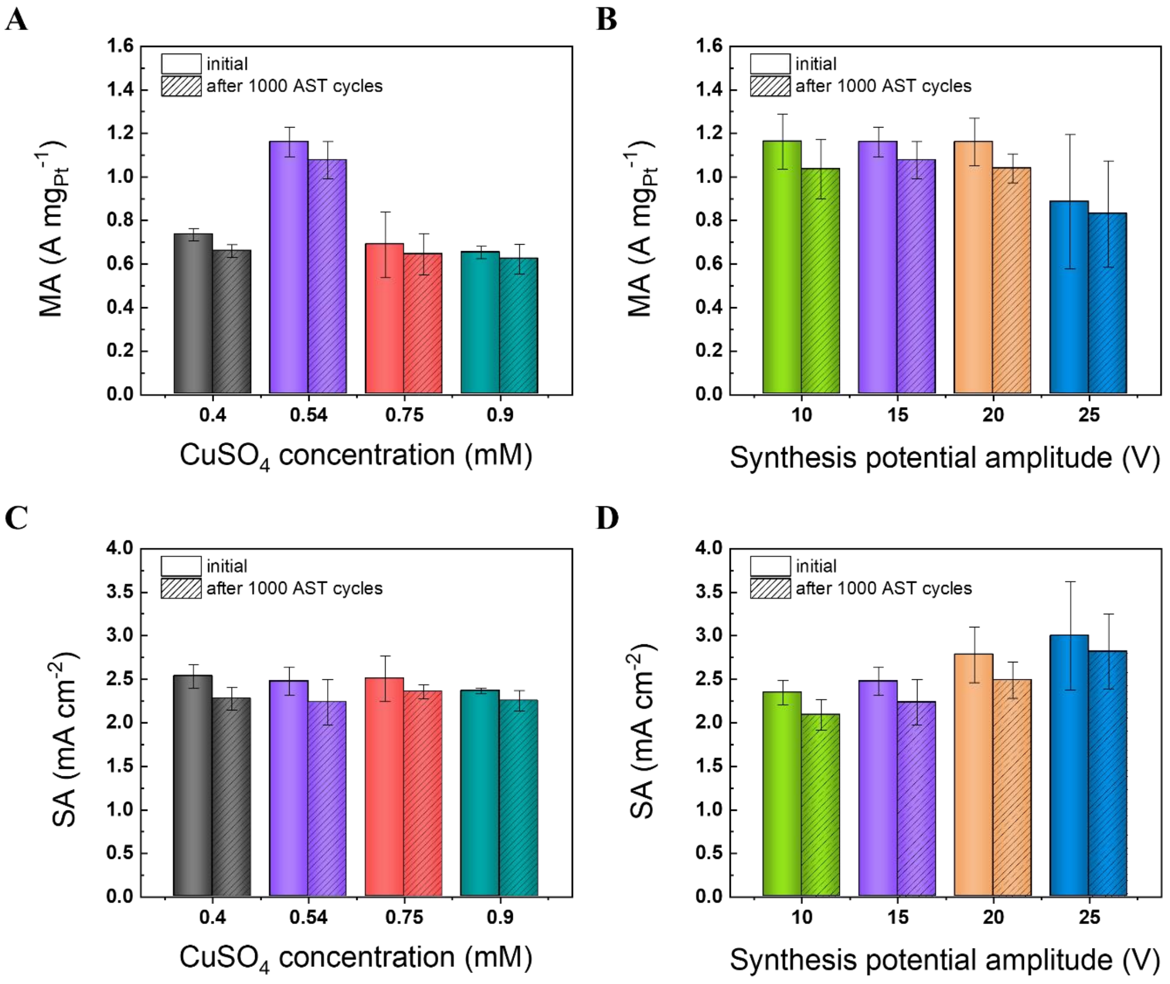

Figure S17. Derived MAs of the synthesized PtCu/C electrocatalysts with (A) varying $CuSO_4$ concentration (0.40 mM – 0.90 mM) and (B) varying potential amplitude (10 V – 25 V). Derived SAs of the synthesized PtCu/C electrocatalysts with (C) varying $CuSO_4$ concentration (0.40 mM – 0.90 mM) and (D) varying potential amplitude (10 V – 25 V). All activities were evaluated at 0.9 V vs. RHE in acidic media (O_2-saturated $HClO_4$).

Activity comparison: Comparing the MA of the produced PtCu/C catalysts in **Figures S17A** and **B**, it becomes apparent that a 0.54 mM $CuSO_4$ concentration yields the maximum MA, while the amplitude of the applied AC potential does not significantly affect the MA. This follows the observed trend of the atomic percentage of Cu (**Figure S1A**), implying that a higher Cu content in the PtCu/C catalyst boosts the MA. However, it seems to be in contradiction to the XRD results presented in **Figure S12A**. According to the Rietveld refinement results, the lattice contraction, i.e., the introduced strain, increases from 0.40 mM to 0.75 mM $CuSO_4$, beyond which it remains constant, as elaborated in the discussion of the XRD results. However, paying attention to the PtCu/C systems in **Figure S17B**, it is evident that all samples with a $CuSO_4$ concentration of 0.54 mM, independently of the synthesis potential amplitude, exhibit

a strongly enhanced MA (~ 1.2 A mg_{Pt}^{-1} at 0.9 V vs. RHE, except for the sample with 25 V being slightly smaller) compared to the samples synthesized with different $CuSO_4$ concentrations (0.40 mM, 0.75 mM, and 0.90 mM). It indicates that the $CuSO_4$ concentration plays a much more significant role in the electrocatalytic performance compared to the potential amplitude, with 0.54 mM being the optimum. In agreement, no clear trend can be observed in the determined lattice contractions for the samples with modified synthesis potential amplitude in **Figure S12B**. Therefore, we conclude that a $CuSO_4$ concentration of 0.54 mM induces an optimal average lattice contraction, i.e., lattice strain, of ~ 1.3 %, optimizing the binding strength between Pt surface sites and ORR intermediates for the electrochemical erosion-derived PtCu/C electrocatalysts. In contrast, higher or lower $CuSO_4$ concentrations lead to more unfavorable average lattice strains (e.g., ~ 1.7 % for $CuSO_4$ concentrations of 075 mM and 0.90 mM). Nevertheless, it should be noted that the XRD results reflect averaged values, since an accurate characterization of the lattice strain of individual nanoparticles is challenging.

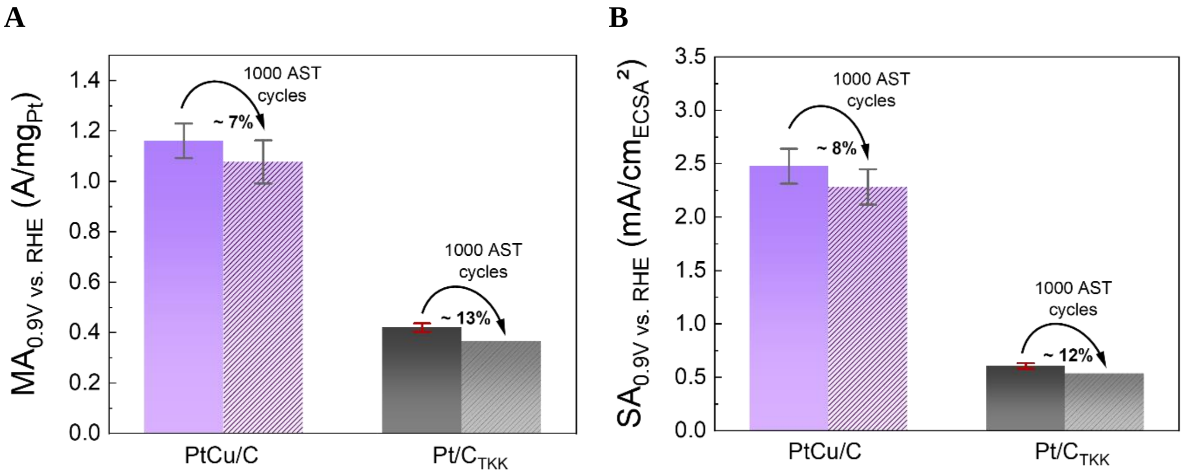

Figure S18. Overview of the ORR (A) MAs and (B) SAs of PtCu/C (0.54 mM $CuSO_4$ concentration + 15 V potential amplitude) and commercial Pt/C_{TKK} (Tanaka) before and after AST. All activities were evaluated at 0.9 V vs. RHE in O_2-saturated 0.1 M $HClO_4$. Stability data of commercial Pt/C_{TKK} was adapted with permission from ref. [7].

Stability interpretation and applicability: As **Figure S18** shows, the PtCu/C catalyst synthesized in this study shows superior stability compared to commercial Pt/C. Nevertheless, it has to be noted that RDE studies do not accurately reflect the conditions during application in MEAs or PEMFCs. However, Fichtner et al. conducted preliminary measurements in a single-cell PEMFC using the Pt/C_{TD} catalyst (~ 20 wt.-% Pt) derived by electrochemical erosion under almost identical synthesis parameters (1 M KOH electrolyte, 200 Hz frequency, and 10

V applied potential amplitude) [7]. MEAs with the Pt/C_{TD} catalyst and with commercial Pt/C_{TKK} (~ 20 wt.-% Pt) were prepared with cathode loadings of ~0.1 mg_{Pt} cm^{-2}. By evaluating the H_2/O_2 polarization curves at 0.9 V, the authors observed a higher MA and SA for the electrochemical erosion-derived Pt/C_{TD}-based MEA. Importantly, they were capable of validating their RDE results in their single-cell PEMFC measurements. This is a strong indication that this study's PtCu/C catalysts could perform in a similar manner, given the almost identical synthetic approach by Fichtner et al., which led to similarly shaped and distributed Pt nanoparticles on carbon compared to our PtCu/C catalysts. Moreover, our best-performing PtCu/C catalysts exhibit even higher MA and SA, which is likely to lead to an even more enhanced PEMFC performance compared to the commercial alternative. Of course, it has to be noted that in the case of PtCu/C, new challenges would emerge, for example, preceding dealloying to avoid contamination of the Nafion membrane and related interfaces with the less noble metal (Cu) through spontaneous dealloying during PEMFC operation [34]. Nevertheless, the preliminary single-cell PEMFC experiment of Fichtner et al. with a pure Pt catalyst derived by electrochemical erosion provides a good estimation for the successful use of this study's PtCu/C catalyst in PEMFCs.

References

[1] J. Fichtner, B. Garlyyev, S. Watzele, H. A. El-Sayed, J. N. Schwämmlein, W. Li, F. M. Maillard, L. Dubau, J. Michalička, J. M. Macak, A. Holleitner, A. S. Bandarenka, Top-Down Synthesis of Nanostructured Platinum–Lanthanide Alloy Oxygen Reduction Reaction Catalysts: PtxPr/C as an Example, ACS Appl. Mater. Interfaces 11 (2019) 5129-5135. https://doi.org/10.1021/acsami.8b20174.

[2] B. Garlyyev, S. Watzele, J. Fichtner, J. Michalička, A. Schökel, A. Senyshyn, A. Perego, D. Pan, H. A. El-Sayed, J. M. Macak, P. Atanassov, I. V. Zenyuk, A. S. Bandarenka, Electrochemical top-down synthesis of C-supported Pt nano-particles with controllable shape and size: Mechanistic insights and application, Nano Res. 14 (2021) 2762-2769. https://doi.org/10.1007/s12274-020-3281-z.

[3] P. Strasser, S. Koh, T. Anniyev, J. Greeley, K. More, C. Yu, Z. Liu, S. Kaya, D. Nordlund, H. Ogasawara, M. F. Toney, A. Nilsson, Lattice-strain control of the activity in dealloyed core–shell fuel cell catalysts, Nature Chem. 2 (2010) 454-460. https://doi.org/10.1038/nchem.623.

[4] Y. Zhao, Y. Wu, J. Liu, F. Wang, Dependent Relationship between Quantitative Lattice Contraction and Enhanced Oxygen Reduction Activity over Pt–Cu Alloy Catalysts, ACS Appl. Mater. Interfaces 9 (2017) 35740-3748. https://doi.org/10.1021/acsami.7b08437.

[5] C. Xia, J. Y. Kim, H. Wang, Recommended practice to report selectivity in electrochemical synthesis of H_2O_2, Nature Catal. 3 (2020) 605-607. https://doi.org/10.1038/s41929-020-0486-1.

[6] R. Zhou, Y. Zheng, M. Jaroniec, S.-Z. Qiao, Determination of the Electron Transfer Number for the Oxygen Reduction Reaction: From Theory to Experiment, ACS Catal. 6 (2016) 4720-4728. https://doi.org/10.1021/acscatal.6b01581.

[7] J. Fichtner, S. Watzele, B. Garlyyev, R. M. Kluge, F. Haimerl, H. A. El-Sayed, W.-J. Li, F. Maillard, L. Dubau, R. Chattot, J. Michalicka, J. M. Macak, W. Wang, D. Wang, T. Gigl, C. Hugenschmidt, A. S. Bandarenka, Tailoring the Oxygen Reduction Activity of Pt Nanoparticles through Surface Defects: A Simple Top-Down Approach, ACS Catal. 10 (2020) 3131-3142. https://doi.org/10.1021/acscatal.9b04974.

[8] M. Zhang, Y. Dai, J.-Y. Hu, S. Miao, B.-Q. Xu, Solvothermal Synthesis of Nanostructured Pt_nNi Tetrahedrons with Enhanced Platinum Utilization and Activity toward Oxygen Reduction Electrocatalysis, J. Phys. Chem. C 125 (2021) 27199-27206. https://doi.org/10.1021/acs.jpcc.1c07755.

[9] A. Matošin, L. Bijelić, A. R. Kamšek, G. Dražić, M. Gatalo, M. Bele, N. Hodnik, Nanoscale Degradation Study of a Commercial Pt–Co/C Fuel-Cell Electrocatalyst at Elevated Temperature Utilizing Identical-Location Scanning Transmission Electron Microscopy, J. Phys. Chem. C 129 (2025) 15419–15432. https://doi.org/10.1021/acs.jpcc.5c03548.

[10] M. Gatalo, P. Jovanovič, U. Petek, M. Šala, V. S. Šelih, F. Ruiz-Zepeda, M. Bele, N. Hodnik, M. Gaberšček, Comparison of Pt–Cu/C with Benchmark Pt–Co/C: Metal Dissolution and Their Surface Interactions, ACS Appl. Energy Mater. 2 (2019) 3131–3141. https://doi.org/10.1021/acsaem.8b02142.

[11] D. Wang, Y. Yu, J. Zhu, S. Liu, D. A. Muller, H. D. Abruña, Morphology and Activity Tuning of Cu_3Pt/C Ordered Intermetallic Nanoparticles by Selective Electrochemical Dealloying, Nano Lett. 15 (2015) 1343-1348. https://doi.org/10.1021/nl504597j.

[12] M. Oezaslan, F. Hasché, P. Strasser, $PtCu_3$, PtCu and Pt_3Cu Alloy Nanoparticle Electrocatalysts for Oxygen Reduction Reaction in Alkaline and Acidic Media, J. Electrochem. Soc. 159 (2012) 444-454. https://doi.org/10.1149/2.106204jes.
[13] M. Wang, W. Zhang, J. Wang, A. Minett, V. Lo, H. Liu, Mesoporous hollow PtCu nanoparticles for electrocatalytic oxygen reduction reaction, J. Chen, J. Mater. Chem. A. 1 (2013) 2391-2394. https://doi.org/10.1039/C2TA01470J.
[14] X. Zhao, H. Cheng, L. Song, L. Han, R. Zhang, G. Kwon, L. Ma, S. Ehrlich, A. I. Frenkel, J. Yang, K. Sasaki, H. L. Xin, Rhombohedral Ordered Intermetallic Nanocatalyst Boosts the Oxygen Reduction Reaction, ACS Catal. 11 (2021) 184-192. https://doi.org/10.1021/acscatal.0c04021.
[15] X. Ye, R.-Y. Shao, P. Yin, H.-W. Liang, Y.-X. Chen, Ordered Intermetallic PtCu Catalysts Made from Pt@Cu Core/Shell Structures for Oxygen Reduction Reaction, Inorg. Chem. 61 (2022) 15239-15246. https://doi.org/10.1021/acs.inorgchem.2c02501.
[16] D. Wang, Y. Yu, H. L. Xin, R. Hovden, P. Ercius, J. A. Mundy, H. Chen, J. H. Richard, D. A. Muller, F. J. DiSalvo, H. D. A Abruña, Tuning Oxygen Reduction Reaction Activity via Controllable Dealloying: A Model Study of Ordered Cu_3Pt/C Intermetallic Nanocatalysts, Nano Lett. 12 (2012) 5230-5238. https://doi.org/10.1021/nl302404g.
[17] N. Hodnik, C. Jeyabharathi, J. C. Meier, A. Kostka, K. L. Phani, . Rečnik, M. Bele, S. Hočevar, M. Gaberščeк, K. J. J. Mayrhofer, Effect of ordering of $PtCu_3$ nanoparticle structure on the activity and stability for the oxygen reduction reaction, Phys. Chem. Chem. Phys. 16 (2014) 13610-13615. https://doi.org/10.1039/C4CP00585F.
[18] M. Bele, P. Jovanovič, A. Pavlišič, B. Jozinović, M. Zorko, A. Rečnik, E. Chernyshova, S. Hočevar, N. Hodnik, M. Gaberšček, A highly active $PtCu_3$ intermetallic core–shell, multilayered Pt-skin, carbon embedded electrocatalyst produced by a scale-up sol–gel synthesis, Chem. Commun. 50 (2014) 13124-13126. https://doi.org/10.1039/C4CC05637J.
[19] V. V. Pryadchenko, S. V. Belenov, D. B. Shemet, V. V. Srabionyan, L. A. Volochaev, A. S. Mikheykin, K. E. Bdoyan, I. Zizak, V. E. Guterman, L. A. Bugaev, Effect of Thermal Treatment on the Atomic Structure and Electrochemical Characteristics of Bimetallic PtCu Core–Shell Nanoparticles in PtCu/C Electrocatalysts, J. Phys. Chem. C. 122 (2018) 17199-17210. https://doi.org/10.1021/acs.jpcc.8b03696.
[20] R. Srivastava, P. Mani, N. Hahn, P. Strasser, Efficient Oxygen Reduction Fuel Cell Electrocatalysis on Voltammetrically Dealloyed Pt–Cu–Co Nanoparticles, Angew. Chem. Int. Ed. 46 (2007) 8988-8991. https://doi.org/10.1002/anie.200703331.
[21] S. Koh, P. Strasser, Electrocatalysis on Bimetallic Surfaces: Modifying Catalytic Reactivity for Oxygen Reduction by Voltammetric Surface Dealloying, J. Am. Chem. Soc. 129 (2007) 12624-12625. https://doi.org/10.1021/ja0742784.
[22] W. Tu, W. Luo, C. Chen, K. Chen, E. Zhu, Z. Zhao, Z. Wang, T. Hu, H. Zai, X. Ke, M. Sui, P. Chen, Q. Zhang, Q. Chen, Y. Li, Y. Huang, 2020. Tungsten as "Adhesive" in $Pt_2CuW_{0.25}$ Ternary Alloy for Highly Durable Oxygen Reduction Electrocatalysis. Adv. Funct. Mater. 30, 1908230. https://doi.org/10.1002/adfm.201908230.
[23] A. A. Alekseenko, A. S. Pavlets, S. V. Belenov, O. I. Safronenko, I. V. Pankov, V. E. Guterman, 2022. The electrochemical activation mode as a way to exceptional ORR performance of nanostructured PtCu/C materials . Appl. Surf. Sci. 595, 153533. https://doi.org/10.1016/j.apsusc.2022.153533.

[24] X. Zhang, Z. An, Z. Xia, H. Li, X. Xu, S. Yu, S. Wang, G. Sun, 2023. Phosphoric acid resistance PtCu/C oxygen reduction reaction electrocatalyst for HT-PEMFCs: A theoretical and experimental study. Appl. Surf. Sci. 619, 156663. https://doi.org/10.1016/j.apsusc.2023.156663.

[25] A. S. Pavlets, A. A. Alekseenko, A. V. Nikolskiy, A. T. Kozakov, O. I. Safronenko, I. V. Pankov, V. E. Guterman, Effect of the PtCu/C electrocatalysts initial composition on their activity in the de-alloyed state in the oxygen reduction reaction, Int. J. Hydrog. Energy 47 (2022) 30460-30471. https://doi.org/10.1016/j.ijhydene.2022.07.014.

[26] H.Cao, J. Cao, F. Wang, S. Di, H. Zhu, M. Pu, A. Bulanova, Composition-tunable PtCu porous nanowires as highly active and durable catalyst for oxygen reduction reaction, Int. J. Hydrog. Energy 46 (2021) 18284-18293. https://doi.org/10.1016/j.ijhydene.2021.02.208.

[27] J. A. Wittkopf, J. Zheng, Y. Yan, High-Performance Dealloyed PtCu/CuNW Oxygen Reduction Reaction Catalyst for Proton Exchange Membrane Fuel Cells, ACS Catal. 4 (2014) 3145-3151. https://doi.org/10.1021/cs500692y.

[28] L. Guo, L.-B. Huang, W.-J. Jiang, Z.-D. Wei, L.-J. Wan, J.-S. Hu, Tuning the branches and composition of PtCu nanodendrites through underpotential deposition of Cu towards advanced electrocatalytic activity, J. Mater. Chem. A 5 (2017) 9014-9021. https://doi.org/10.1039/C7TA01859B.

[29] J. Ying, G. Jiang, Z. P. Cano, Z. Ma, Z. Chen, Spontaneous weaving: 3D porous PtCu networks with ultrathin jagged nanowires for highly efficient oxygen reduction reaction, Appl. Catal. B: Environ. 236 (2018) 359-367. https://doi.org/10.1016/j.apcatb.2018.04.035.

[30] B.-A. Lu, T. Sheng, N. Tian, Z.-C. Zhang, C. Xiao, Z.-M. Cao, H.-B. Ma, Z.-Y. Zhou, S.-G. Sun, Octahedral PtCu alloy nanocrystals with high performance for oxygen reduction reaction and their enhanced stability by trace Au, Nano Energy 33 (2017) 65-71. https://doi.org/10.1016/j.nanoen.2017.01.003.

[31] S. Luo, M. Tang, P. K. Shen, S. Ye, 2017. Atomic-Scale Preparation of Octopod Nanoframes with High-Index Facets as Highly Active and Stable Catalysts. Adv. Mater. 29, 1601687. https://doi.org/10.1002/adma.201601687.

[32] H. Y. Kim, T. Kwon, Y. Ha, M. Jun, H. Baik, H. Y. Jeong, H. Kim, K. Lee, S. H. Joo, Intermetallic PtCu Nanoframes as Efficient Oxygen Reduction Electrocatalysts, Nano Lett. 20 (2020) 7413-7421. https://doi.org/10.1021/acs.nanolett.0c02812.

[33] M. Gong, D. Xiao, Z. Deng, R. Zhang, W. Xia, T. Zhao, X. Liu, T. Shen, Y. Hu, Y. Lu, X. Zhao, H. Xin, D. Wang, 2021. Structure evolution of PtCu nanoframes from disordered to ordered for the oxygen reduction reaction. Appl. Catal. B: Environ. 282, 119617. https://doi.org/10.1016/j.apcatb.2020.119617.

[34] F. Zhu, A. Wu, L. Luo, C. Wang, F. Yang, G. Wei, G. Xia, J. Yin, J. Zhang, The Asymmetric Effects of Cu^{2+} Contamination in a Proton Exchange Membrane Fuel Cell (PEMFC), Fuel Cells 20 (2020) 196-202. https://doi.org/10.1002/fuce.201900189.
